# Biomarkers for predicting immunotherapy response and resistance in glioblastoma

**DOI:** 10.3389/fimmu.2026.1823338

**Published:** 2026-05-05

**Authors:** Samuel Kim, Matthew Abikenari, Brandon Bergsneider, Lily H. Kim, Michael Lim

**Affiliations:** 1 University of Missouri-Kansas City School of Medicine, University of Missouri-Kansas City, Kansas City, MO, United States; 2 Stanford University School of Medicine, Stanford University, Stanford, CA, United States; 3 Department of Neurosurgery, Stanford University School of Medicine, Stanford University, Stanford, CA, United States

**Keywords:** artificial intelligence, biomarkers, glioblastoma, immunotherapy, interferon signaling, neuro-immunology

## Abstract

Glioblastoma is the most common and aggressive primary brain tumor in adults that fails to meet survival endpoint criteria in current immunotherapy Phase III clinical trials. As a small subset of immunotherapy-responsive patients has been identified, biomarkers that can nominate patients for immunotherapy and inform treatment combinations have been increasingly urgent. Mechanistically-driven single biomarkers (e.g. PD-1/PD-L1 enrichment, tumor mutational burden, and *MGMT* methylation) are early opportunities for immunotherapy response prediction but have had inconsistent results across studies. In contrast, new context-dependent biomarker signals such as interferon signaling, immune cell population studies, and radiographic biomarkers show promise in vaccine-based therapies and in immune checkpoint blockade. Ongoing clinical trials are increasingly implementing early exploratory endpoints for biomarker discovery from several body compartments (e.g. CSF, blood, tissue). Present advancements in machine learning and liquid biopsy have provided nuanced biomarker signatures that can be composed of several biomarker categories. Progress in standardizing biomarkers in glioblastoma immunotherapy relies on the early implementation and uniformity of biomarker-driven endpoints in clinical trials.

## Introduction

Glioblastoma (GBM) is the most common and aggressive primary malignant brain tumor in adults, with a median survival of 12–18 months despite treatment (1). The current standard of care (SOC) of resection followed by radiotherapy (RT) and temozolomide, known as the Stupp protocol, remains unchanged for over two decades (2). The intrinsic therapeutic resistance of GBM is driven by a multitude of collaborating factors including tumor-associated immunosuppression (3, 4), stromal and cellular components of resistance (e.g. myeloid derived suppressor cells, tumor-infiltrating lymphocytes, and fibroblasts) (4), corruption of the blood brain barrier (5), and the emerging neuron-glioma axis (6, 7). Efforts to therapeutically target individual resistance pathways have been difficult due to intra- and inter-tumoral heterogeneity (8, 9).

Unlike other solid tumors such as melanoma and non-small cell lung cancer (10–12), GBM remains resistant across various immune checkpoint blockades (ICB) in clinical trials despite response to anti-PD-1 therapy and radiation in murine models (13). Adoptive T cell therapies, cancer vaccines, and oncolytic viruses have shown promise in preclinical settings as well, but clinical trial outcomes in GBM have been disappointing. There are reports of outlier cases in which GBM patients exhibit durable responses to immunotherapy (14–19). The absence of validated biomarkers poses a central barrier to predicting immunotherapy response and delivering precision therapies to responsive patients (20, 21).

Indeed, as immunotherapy trials in GBM expand, biomarker discovery is becoming increasingly critical. Several candidate biomarkers of immunotherapy response have been proposed (**Table 1**), but most exist in isolation and lack systematic validation (46). As a result, the clinical utility of most biomarkers remains uncertain at present despite efforts to incorporate biomarkers into trial designs. In this review, biomarkers for GBM responsiveness and resistance to immunotherapy are broadly discussed in order to recapitulate the current progress in biomarker nomination. The review aims to provide frameworks for future testing and implementation of biomarkers in clinical workflows instead of being an exhaustive list of biomarkers. In this review, biomarkers are described as a “defined characteristic that is measured as an indicator of normal biological processes, pathogenic processes or responses to an exposure or intervention, including therapeutic interventions” as determined by the FDA–NIH Biomarker Working Group (47). By doing so, this review attempts to map the current landscape of translationally relevant biomarker candidates and lay groundwork for their systematic evaluation in future clinical investigation.

**Table 1:** Modality-specific biomarkers of response and resistance across GBM immunotherapy platforms.

Modality and agent class	Biomarker (reference)	Biomarker type	Sample source	Assay/readout	Association with response	Timing	Level of evidence	Mechanistic rationale	Major limitations
ICB	PD-L1 expression (22)	Predictive biomarker	Tumor	IHC	Mixed predictive value; prognostic in some GBM cohorts; not validated for response selection	Pre-treatment	Retrospective, heterogeneous in clinical trials	Reflects adaptive immune resistance signaling	Intratumoral heterogeneity and assay variability; lacks validated predictive threshold in GBM
ICB	Tumor mutational burden (23)	Predictive biomarker	Tumor	NGS	Generally not predictive in GBM; high TMB rare	Pre	Mixed	Neoantigen load concept not consistently linked to GBM response	Low baseline TMB in GBM; poor correlation with neoantigen immunogenicity
ICB	dMMR	Predictive biomarker	Tumor	IHC/NGS	May be associated with response in rare hypermutated cases	Pre	Case-level	Hypermutation	Rare in GBM; limited generalizability beyond hypermutated subsets
ICB	MAPK pathway alterations (24)	Predictive biomarker	Tumor	NGS	Enriched among responders in some recurrent GBM cohorts	Pre	Retrospective + preclinical	Oncogenic signaling affects immune sensitivity	Derived from small retrospective cohorts; lacks prospective validation
ICB	Activated pERK1/2 (25)	Predictive biomarker	Tumor	Phospho-IHC	Higher activation associated with improved response and survival in reports	Pre/on	Retrospective	MAPK activity linked to PD-1 sensitivity	Limited reproducibility; lacks prospective validation
ICB	IFN-gamma gene signature (26)	Pharmacodynamic/ prognostic/ predictive biomarker	Tumor	RNA-seq	Higher expression associated with longer survival in some ICB settings	Pre/on	Clinical correlative	Inflamed tumor state	Context-dependent effects; overlaps with both immune activation and exhaustion states
ICB	PTEN alterations (27)	Predictive biomarker	Tumor	NGS	Enriched in non-responders	Pre	Retrospective	Immune exclusion programs	Broad downstream effects; not specific to immune resistance mechanisms; lacks prospective validation
ICB	Cell-free DNA (28)	Predictive biomarker	CSF/ Blood/ Interstitial fluid	Cell-free DNA/NGS	Dynamic changes associated with disease course	Longitudinal	Proof-of-concept	Tumor burden tracking	Low tumor shedding in GBM and blood–brain barrier limits sensitivity
OV therapy	Baseline ISG (29)	Predictive/ pharmacodynamic biomarker	Tumor	RNA-seq	Higher ISG associated with reduced OV efficacy	Pre	Early + preclinical	Antiviral state	Spatial and temporal heterogeneity; lacks prospective validation and standardized thresholds.
DC vaccine	MGMT methylation (30)	Prognostic/ predictive biomarker	Tumor	Methylation	Associated with improved OS and PFS	Pre	Phase II/III	Baseline tumor biology	Primarily prognostic; not specific to immunotherapy response
DC vaccine	Extent of resection (31)	Prognostic biomarker	Clinical	Operative	Greater resection associated with improved outcomes	Pre	Clinical trials	Lower tumor burden	Not a molecular biomarker; confounded by surgical and patient selection factors
DC vaccine	HLA-A2/A1 status (32)	Predictive biomarker	Germline	HLA typing	Associated with benefit in defined vaccine subgroups	Pre	Phase II	Antigen presentation	Limited applicability to specific vaccine constructs; not generalizable
DC vaccine	Peripheral CD8 count (33)	Predictive/prognostic biomarker	Blood	Flow	Higher counts associated with longer OS	Pre	*Post hoc*	Immune competence	Nonspecific immune marker; influenced by systemic factors such as corticosteroids
DC vaccine	Peripheral monocytes (33)	Predictive/prognostic biomarker	Blood	Flow	Higher counts associated with longer OS	Pre	*Post hoc*	APC availability	Poor specificity; can be influenced by systematic factors such as corticosteroids
DC vaccine	Treg levels (33)	Predictive/prognostic biomarker	Blood	Flow	Higher levels associated with worse survival	Pre/on	*Post hoc*	Immune suppression	Dynamic and treatment-sensitive; can be affected by corticosteroids and biological sex
DC vaccine	PD-1+/CD8 ratio (34)	Prognostic biomarker	Blood/tumor	Flow/IHC	Higher ratios associated with worse outcomes	Pre	*Post hoc*	Exhaustion burden	Reflects exhaustion but lacks validated predictive thresholds
DC vaccine	IFN-gamma ELISPOT (33)	Prognostic/ pharmacodynamic biomarker	PBMC	ELISPOT	Higher post-vaccine response associated with improved OS/PFS	On	Correlative	Functional T-cell activity	Assay variability and lack of standardized cutoffs; limited cross-study comparability
DC vaccine	CTLA-4 CD8 ratio (35)	Predictive/prognostic biomarker	Blood	Flow	Lower ratios associated with longer OS	On	Correlative	Reduced inhibition	Exploratory metric with limited reproducibility across cohorts
HSP vaccine	Low myeloid PD-L1 (36)	Predictive/prognostic biomarker	Blood	Flow	Lower expression associated with improved survival	Pre	Phase II correlative	Reduced myeloid suppression	Dynamic and compartment-dependent expression; limited standardization of myeloid cell phenotyping and uncertain specificity for vaccine-induced response
HSP vaccine	TSIR (37)	Predictive/prognostic biomarker	PBMC	ELISPOT	Higher post-vaccine TSIR associated with improved PFS	On	Phase I/II	Vaccine-induced immunity	Assay-dependent variability; limited standardization across studies
HSP vaccine	TCR diversity (38)	Predictive/prognostic biomarker	Tumor/blood	TCR-seq	Lower diversity associated with longer survival in reports	Pre/on	Correlative	Clonal expansion	Context-dependent interpretation; insufficient evidence for clinical implementation
HSP vaccine	MxA (39)	Predictive/prognostic/ pharmacodynamic biomarker	Blood	Expression	Lower expression associated with long-term survival	Pre	Emerging	Interferon proxy	Indirect interferon marker; lacks specificity for treatment response
WT1 vaccine	WT1 expression (40)	Predictive biomarker	Tumor	IHC	Higher expression associated with improved OS/PFS	Pre	*Post hoc*	Target abundance	Target expression does not ensure effective immune recognition or response; therapy specific
WT1 vaccine	SDC-4 (41)	Predictive/prognostic biomarker	Blood	qPCR	Higher expression associated with worse OS	Pre	Emerging	Immune signaling	Limited validation; unclear mechanistic role in immunotherapy response
Exploratory	Extracellular vesicles (42)	Predictive/prognostic biomarker	CSF/ Blood	Vesicle isolation and cargo profiling	Dynamic changes in EV cargo are associated with treatment response and disease progression; predictive utility remains unvalidated	Longitudinal	Exploratory	Tumor heterogeneity and communication	Lack of standardized isolation and assay methods; exploratory evidence as of now
CAR T	EGFRvIII (43)	Predictive biomarker	Tumor	IHC/NGS	Required for eligibility	Pre	Early clinical	Target presence	Heterogeneous and unstable expression; antigen loss under therapeutic pressure; therapy specific
Imaging	rADC post-ICI (44)	Predictive/prognostic biomarker	MRI	ADC	Higher post-ICI rADC associated with longer survival	Post	Retrospective	Diffusion reflects response biology	Susceptible to pseudoprogression and treatment-related imaging changes; not confirmed in prospective studies
Radiomics	Radiomic TIME models (45)	Predictive/prognostic/ pharmacodynamic biomarker	MRI	ML	Associated with immune infiltration patterns	Pre	Retrospective	Imaging proxy	Lack of external validation and susceptibility to overfitting in small datasets; lacks prospective validation

This table summarizes candidate biomarkers evaluated across immune checkpoint blockade (ICB), dendritic cell (DC) vaccination, heat shock protein (HSP) vaccines, peptide vaccines, oncolytic viral (OV) therapy, chimeric antigen receptor (CAR) T-cell therapy, liquid biopsy, and radiographic approaches in GBM. Biomarkers are organized by mechanistic axis, type of biomarker, specimen source, assay modality, timing relative to therapy, level of supporting evidence, and the major limitations to clinical implementation.

GBM, glioblastoma; ICB, immune checkpoint blockade; PD, pharmacodynamic; PD-1, programmed cell death protein 1; PD-L1, programmed death-ligand 1; CTLA-4, cytotoxic T-lymphocyte–associated protein 4; LAG-3, lymphocyte activation gene 3; TIM-3, T-cell immunoglobulin and mucin domain–containing protein 3; TILs, tumor-infiltrating lymphocytes; TMB, tumor mutational burden; dMMR, deficient mismatch repair; MAPK, mitogen-activated protein kinase; ERK, extracellular signal–regulated kinase; pERK1/2, phosphorylated extracellular signal–regulated kinase 1/2; IFN-γ, interferon gamma; ISG, interferon-stimulated gene; IDO1, indoleamine 2,3-dioxygenase 1; PTEN, phosphatase and tensin homolog; DC, dendritic cell; APC, antigen-presenting cell; HLA, human leukocyte antigen; OS, overall survival; PFS, progression-free survival; Treg, regulatory T cell; PBMC, peripheral blood mononuclear cell; ELISPOT, enzyme-linked immunospot assay; HSP, heat shock protein; TSIR, tumor-specific immune response; TCR, T-cell receptor; WT1, Wilms tumor 1; SDC-4, syndecan-4; CSF, cerebrospinal fluid; ctDNA, circulating tumor DNA; CAR, chimeric antigen receptor; EGFR, epidermal growth factor receptor; EGFRvIII, epidermal growth factor receptor variant III; MRI, magnetic resonance imaging; rADC, relative apparent diffusion coefficient; ADC, apparent diffusion coefficient; ML, machine learning; TIME, tumor immune microenvironment; NGS, next-generation sequencing; IHC, immunohistochemistry; qPCR, quantitative polymerase chain reaction.

## Section 1. Conceptual framework for biomarker evaluation in glioblastoma immunotherapy

### Biomarker classification and scope

Several categories of biomarkers exist including diagnostic, monitoring, predictive, prognostic, and response biomarkers–typically split into pharmacodynamic and surrogate endpoint biomarkers (48). Of these taxonomical categories, prognostic, predictive, and pharmacodynamic biomarkers are central to the discovery, development, and widespread clinical integration of novel drugs in cancer medicine (49, 50). Prognostic biomarkers are defined as measures that identify the likelihood of a clinical event–most commonly survival endpoints–in patients with a medical condition. Predictive biomarkers are defined as measures that identify the likelihood that an individual in a cohort of similar individuals will experience a favorable or unfavorable effect from an exposure. Finally, pharmacodynamic biomarkers are defined as measures of biological activity of an agent without determining specific effects (48). This review centers on predictive biomarkers of immunotherapy response in GBM, with supplementary discussion of prognostic and pharmacodynamic markers, while recognizing that these categories frequently overlap in the existing literature (48).

Predictive biomarkers are quantifiable measurements that anticipate tumor responsiveness to immunotherapy. In GBM, responsiveness is often defined by the Response Assessment in Neuro-Oncology (RANO) 2.0 criteria (51) or intratumoral immunogenicity (26). Survival has also been used to indicate response–thereby, describing a biomarker that is both predictive and prognostic (48, 52). Defining immunotherapy responsiveness is both difficult and heterogeneous across the literature, fraught with challenges in both sequential biopsy for tissue analysis and pseudoprogression on magnetic resonance imaging (MRI) (8, 53, 54). As seen in **Table 2**, proper biomarker identification for immunotherapy responsiveness begins with investigation of mechanisms for immunotherapy resistance in GBM to generate mechanism-driven, predictive biomarkers (55). Once predictive biomarkers are nominated, careful decisions must be made regarding endpoints and measures for recording tumor responsiveness to immunotherapy.

**Table 2:** Multimodal biomarker framework linking resistance mechanisms to measurement and action.

Resistance mechanism	Compartment	Example biomarkers	Best measurement	When	Primary use	Key limitation
Checkpoint dominance and T-cell exhaustion	TIME/systemic	PD-1, PD-L1, LAG-3, TIM-3, PD-1/CD8 ratios	IHC + flow + RNA signatures	Baseline + early on-treatment	Combination selection; PD monitoring	Assay cutoffs vary
Antigen presentation loss	Tumor	MHC-I reduction, HLA constraints, antigen loss	IHC + NGS + spatial	Baseline	Vaccine/CAR-T eligibility	Spatial heterogeneity
Tumor oncogenic immune sculpting	Tumor	EGFR, EGFRvIII, PTEN loss, MAPK activation	NGS + phospho-IHC	Baseline	Stratification; combinatorial design	Often correlative
Interferon-axis state	Tumor/TIME	Type I ISG, IFN-gamma signatures, MxA	RNA-seq + protein	Baseline + on-treatment	Therapy selection; OV candidacy	Strong context dependence
Myeloid and MDSC dominance	TIME/systemic	CD163, CSF1R, CD33, monocytes	IHC + flow + deconvolution	Baseline + longitudinal	Myeloid-targeted combinations	Steroid confounding
Hypoxia–angiogenesis axis	TIME/imaging	HIF1α, VEGF, perfusion features	IHC + MRI perfusion	Baseline	Anti-angiogenic pairing	Regional variability
Metabolic suppression	TIME/systemic	IDO1, kynurenine pathways	IHC + metabolomics	Baseline/on	Metabolic combinations	Systemic confounders
Functional anti-tumor immunity	Systemic	ELISPOT, TSIR, IL-2 responsiveness	PBMC functional assays	On-treatment	Pharmacodynamic readout	Assay variability
Immune repertoire constraints	Tumor/systemic	TCR diversity, clonotypes	TCR-seq	Baseline/on	Stratification	Interpretation varies
Radiographic immune proxies	Imaging	ADC, rCBV, radiomic immune scores	MRI radiomics	Baseline + post	Noninvasive monitoring	Protocol variability
Liquid biopsy longitudinal	CSF/blood	cfDNA methylation, immune panels, extracellular vesicles	ctDNA + flow	Longitudinal	Monitoring	Sensitivity limits
AI composite signatures	Integrative	Multimodal composite scores	ML integration	Prospective	Enrollment risk score	Needs external validation

This table outlines a conceptual framework aligning major resistance mechanisms in GBM immunotherapy with representative biomarkers, optimal measurement platforms, timing of assessment, and potential clinical applications. The table includes key limitations and primary uses of the proposed biomarkers thematically.

GBM, glioblastoma; TIME, tumor immune microenvironment; PD-1, programmed cell death protein 1; PD-L1, programmed death-ligand 1; LAG-3, lymphocyte activation gene 3; TIM-3, T-cell immunoglobulin and mucin domain–containing protein 3; MHC-I, major histocompatibility complex class I; HLA, human leukocyte antigen; EGFR, epidermal growth factor receptor; EGFRvIII, epidermal growth factor receptor variant III; PTEN, phosphatase and tensin homolog; MAPK, mitogen-activated protein kinase; ISG, interferon-stimulated gene; IFN-γ, interferon gamma; MxA, myxovirus resistance protein A; MDSC, myeloid-derived suppressor cell; CSF1R, colony-stimulating factor 1 receptor; HIF1α, hypoxia-inducible factor 1 alpha; VEGF, vascular endothelial growth factor; IDO1, indoleamine 2,3-dioxygenase 1; ELISPOT, enzyme-linked immunospot assay; TSIR, tumor-specific immune response; IL-2, interleukin-2; PBMC, peripheral blood mononuclear cell; TCR, T-cell receptor; MRI, magnetic resonance imaging; ADC, apparent diffusion coefficient; rCBV, relative cerebral blood volume; cfDNA, cell-free DNA; CSF, cerebrospinal fluid; ML, machine learning; NGS, next-generation sequencing; IHC, immunohistochemistry.

### Criteria for biomarker candidacy in glioblastoma immunotherapy

Despite increasing studies in biomarkers, across the literature, less than 1% of published biomarkers enter clinical practice (56). Biomarkers in cancer medicine are often technically valid but do not hold clinical utility (57, 58). Therefore, an ideal nominated biomarker should have clinical validity and utility while remaining feasible and safe during implementation (59). The analyte or feature studied as a biomarker should have biological plausibility and measure standardization (48). Clinical utility is the capacity for a predictive biomarker to improve patient outcomes after clinical implementation (60). For GBM immunotherapy, the idealized biomarker would pragmatically improve patient outcomes through patient-treatment stratification, specifically in clinical trials.

Nominations for predictive biomarkers in cancer medicine should be approached cautiously and involve a systematic method to bring retrospectively and bioinformatically identified candidates to clinical trials (61, 62). The key criterion for advancing nominated analytes is whether the proposed predictive biomarker has a plausible and measurable impact on improving the current standard of care (61). Robust evidence for a decidedly promising predictive biomarker should be thoroughly tested in multiple, isolated clinical trials (63). Clinical trials that include biomarker discovery should report the findings with detailed rationale, uniform assays and read outs, careful patient selection, appropriate study design, and statistical validity (64). Once validated in at least two clinical trials (63), confirmed biomarkers must demonstrate clinical feasibility and cost effectiveness (59). In this review, predictive biomarkers across the five phases of biomarker discovery are discussed including the weakest evidence (e.g. bioinformatic nominations and preclinical studies) to stronger levels of evidence (e.g. retrospective longitudinal and prospective trials) (65).

### Compartments for biomarker procurement in glioblastoma immunotherapy

Predictive biomarkers for GBM immunotherapy can be obtained from several compartments including tissue parenchyma, cerebrospinal fluid (CSF), and blood (8). Earliest trials in GBM immunotherapy utilized tissue sampling and retrospective analysis to identify molecular biomarkers such as Programmed Cell Death Protein 1 (PD-1) levels after ICB (66, 67). Indeed, tissue sampling remains the most utilized compartment for procurement of biomarkers to evaluate in GBM immunotherapy (68). Tissue sampling enables comprehensive characterization of the heterogeneous GBM tumor microenvironment, including immunophenotype, molecular markers (e.g., PD-1/PD-L1, tumor antigens), genetic features, and cytokine profiles (55). However, tissue sampling via repeat biopsy carries substantial risk for patients with GBM (8) and cannot be done longitudinally.

Liquid biopsies provide a new compartment for safe retrieval of tissue for biomarker discovery (69). Conventionally, liquid biopsies capture the retrieval of patient peripheral blood, urine, or CSF (70, 71). Liquid biopsies provide analytes such as cell-free DNA (cfDNA), circulating RNA (circRNA), circulating tumor cells (CTC), peripheral blood mononuclear cells (PBMC), and extracellular vesicles (EV) (72–76). Liquid biopsies have several advantages over tissue sampling or standard MRI including perioperative monitoring without biopsy availability restrictions (77), characterization of eloquent-area GBM (71), and differentiating true progression and pseudoprogression (78). Each biofluid source has distinct limitations and benefits. CSF samples have significantly higher detection rates of nucleic acids and EVs due to anatomical proximity to the GBM source (79). However, lumbar punctures for CSF collection are not possible in all patients and are more invasive than collecting peripheral blood. Biofluids such as peripheral blood, serum, and plasma are considerably less invasive and ideal for recurring collection and analysis (80) with a tradeoff for detection sensitivity. Other biofluids such as interstitial fluids (81) and urine (82) are early and should continue to be investigated to gauge clinical utility. Despite the advantages of liquid biopsies, barriers remain for clinical translation including society consensus, standardized assays and procedural checklists, and evidence for feasible and cost-effective clinical utility (83). As a result, testing of liquid biopsies–importantly, CSF sampling–must be included in future immunotherapy clinical trials.

## Section 2. Barriers to biomarker discovery and validation in glioblastoma immunotherapy

### Glioblastoma heterogeneity

Early single-cell RNA-sequencing demonstrated GBM heterogeneity between patients and within an individual tumor (70). GBM intratumoral and intertumoral heterogeneity defines the tumor-specific immunotherapy resistance (84, 85), the existence of long-term survivors (86), and the central barrier to implementing universal, FDA-approved predictive biomarkers in GBM immunotherapy clinical trials. Efforts to deconvolute GBM heterogeneity (27) through subtyping (e.g. proneural, neural, classical, and mesenchymal) provide only minimal benefit due to intratumoral heterogeneity (85). As such, there is an overall lack of confidence at the clinical utility of GBM molecular deconvolution in altering the standard of care (87). The barriers for biomarker nomination due to GBM heterogeneity is perpetuated by heterogeneity between newly diagnosed and recurrent GBM (rGBM) (88) and the fluctuating cellular environment due to treatment-related pressures (26). Acquired resistance and tumor genomic and immune remodeling due to immunotherapeutic pressures continues to be ambiguous (24, 84, 89) but explains the lack of uniformity in results for biomarker validation in GBM immunotherapy clinical trials.

### Clinical variables affecting analyte measurement and validity

Corticosteroids are used to treat vasogenic edema in GBM by suppressing Vascular Endothelial Growth Factor (VEGF) and vascular permeability (90). The necessity of corticosteroids are due to both GBM progression and to reduce edematous sequelae of chemoradiation (91). However, corticosteroids reduce GBM immunogenicity through the reduction of proinflammatory molecules (92), decrease peripheral circulating lymphocytes (93, 94), and, therefore, attenuate the efficacy of immunotherapy in GBM (22, 95). In the Checkmate 143 Phase III randomized clinical trial (RCT), baseline prednisone or dexamethasone use was associated with shorter survival in the ICB treatment group (96). Predictive biomarkers reliant on blood biofluids or infiltrating immune populations in tumor samples are confounded by the immunosuppressive effects of corticosteroids and act as a barrier for reliable analyte quantification.

Biological sex plays an important role in GBM survival and immunogenicity (97, 98). In healthy individuals, females are generally accepted to have more responsive immunophenotype with lower numbers of T regulatory cells (Treg), more active T cell repertoire after stimulation, and a greater antibody response (99–102). Preclinical models demonstrate increased T-cell exhaustion in male GBM (103) and female-associated pathogenic microglia suppression (104). A study of the The Cancer Genome Atlas (TCGA) found that female GBM was associated with enrichment of adaptive immune response genetic signatures that significantly predicted survival benefits. Subsequent meta-analysis of clinical trials data showed that female participants had improved survival at 1-year compared to male participants across 10 pooled studies (98). Identification of predictive biomarkers for GBM immunotherapy responsiveness are challenging due to the inherent immunogenic dimorphism between female and male clinical trials participants. For example, a hypothetical predictive biomarker–such as peripheral Treg cells–may not be useful in females despite validity in males. Clinical trial designs must consider the clinical variables that can affect the reliability, uniformity, and clinical utility of nominated and tested biomarkers.

### Challenges in study design

Clinical trials designs for exploring predictive biomarkers for various tissue or biofluid sources have been proposed but remain insufficiently standardized (55). Heterogeneity between immunotherapy type, clinical trial design, assays and cut off values decided, sample sizes, and procurement timeline contribute to the complexities and barriers to biomarker discovery and implementation. Even with standardized trial designs, variability in tissue collection and processing continue to undermine biomarker investigation in clinical trials (105, 106) and future generalizability. Moreover, due to the relative rarity of GBM, small sample sizes are a major barrier to biomarker discovery and implementation because they reduce statistical power, increase the risk of false-positive and false-negative associations, limit subgroup analyzes, and impair generalizability and methods to reduce noise from the inherent GBM heterogeneity. Issues with proper powering of studies may be exacerbated in the recent era of combinatorial therapy study designs requiring multiple study arms based on factorial methodology (55, 106, 107). There is an increasingly urgent necessity for harmonized procedures for trial designs that include recommendations for tissue processing, assays and cut off values, and data harmonization.

## Section 3. Biomarkers of immune checkpoint blockade

### Programmed cell death protein 1/programmed death-ligand 1

The discovery of antibodies targeting endogenous negative regulatory pathways of T-cell activation in solid and metastatic tumors is one of the most important breakthroughs in immunotherapy (108). The most notable examples in GBM include blockade of the Cytotoxic T-Lymphocyte Antigen 4 (CTLA-4) and the Programmed Cell Death Protein 1 (PD-1)/Programmed Death-Ligand 1 (PD-L1) pathways (66, 96). To date, there are no validated predictive biomarkers for anti-CTLA-4 therapy and anti-PD-1/PD-L1 therapy in GBM; however, in other solid tumors, PD-1 and PD-L1 enrichment have been considered as biomarkers for immunotherapy sensitivity due to their physiological role in native and tumorogenic T-cell activation (109). Even still, the evidence for the utility of PD-1/PD-L1 enrichment in all solid tumors remains contentious due to widespread heterogeneity in cut off values and immunohistochemistry (IHC) assays (110). PD-1/PD-L1 enrichment is similarly ambiguous in GBM, with no studies to date confirming the validity of PD-1/PD-L1 enrichment as a predictor for ICB responsiveness (67). In an early study of 135 retrospectively identified formalin-fixed and paraffin-embedded (FFPE) tumor tissue, membranous and diffuse/fibrillary PD-1 expression on immunohistochemistry (IHC) analysis was not associated with infiltrating lymphocyte levels or survival (111). In the KEYNOTE-028 phase I trial of pembrolizumab in PD-L1–positive rGBM, responses were observed across a wide range of PD-L1 expression levels, with the two responders exhibiting tumor PD-L1 expression of 1% and 100%, respectively (67). In the randomized Phase II investigating pembrolizumab plus bevacizumab versus pembrolizumab alone, PD-1 expression in archival samples was not a reliable prognostic and predictive biomarker (22). Although biologically plausible, PD-1/PD-L1 expression alone has not reliably identified ICB responders in GBM in clinical trials and does not fit aforementioned criteria for a reliable predictive biomarker for patient selection.

### Tumor mutational burden

Like PD-1/PD-L1, tumoral mutational burden (TMB) is a mechanism-driven biomarker of interest as TMB is associated with high neoantigen production and, therefore, related to possible ICB responsiveness (112–114). Defective mismatch repair (dMMR) status is also a molecular biomarker of interest as deficiency in mismatch repair is a key driver of oncogenesis through the proliferation of mutations, resulting in increased TMB (115). As such, TMB and MMR status have both been used to stratify possible ICB responses in clinical trials of other cancers (114). Indeed, in pediatric GBM, two siblings with recurrent multifocal biallelic MMR-deficiency GBM were identified and treated with nivolumab with significant clinical and radiological response (16). However, high TMB is found in only 2.7% of GBM and is not a reliable biomarker for CD8+ T-cell enrichment (116). As such, there is insufficient evidence for TMB as a reliable biomarker for GBM due to the rarity for high TMB phenotypes. In *post hoc* exploratory analyzes of a study on high-grade glioma (HGG) in patients who had partial or complete loss of MMR protein expression and were treated with pembrolizumab, high TMB was not an effective biomarker for ICB response (117). Similarly, in an multinational, open-label, phase 2 study investigating PD-L1 blockade with durvalumab with or without bevacizumab, high TMB was not related to progression free survival (PFS) in both treatment arms (118). Paradoxically, low TMB has been found to predict responsiveness–defined as survival time–to both virotherapy and ICB therapy in rGBM (23). The exact physiological mechanism for this relationship has yet to be determined but one hypothesis is that TMB is limited in patients with functional immune surveillance, and it is those patients with immunologically active tumors that respond the best to ICB. Although further evaluation of TMB in well-powered clinical trials is needed to determine its predictive or prognostic value, the requirement for substantial tissue input for accurate TMB assessment may limit its clinical utility (22).

### MAPK/ERK pathway

**Figure 1:**
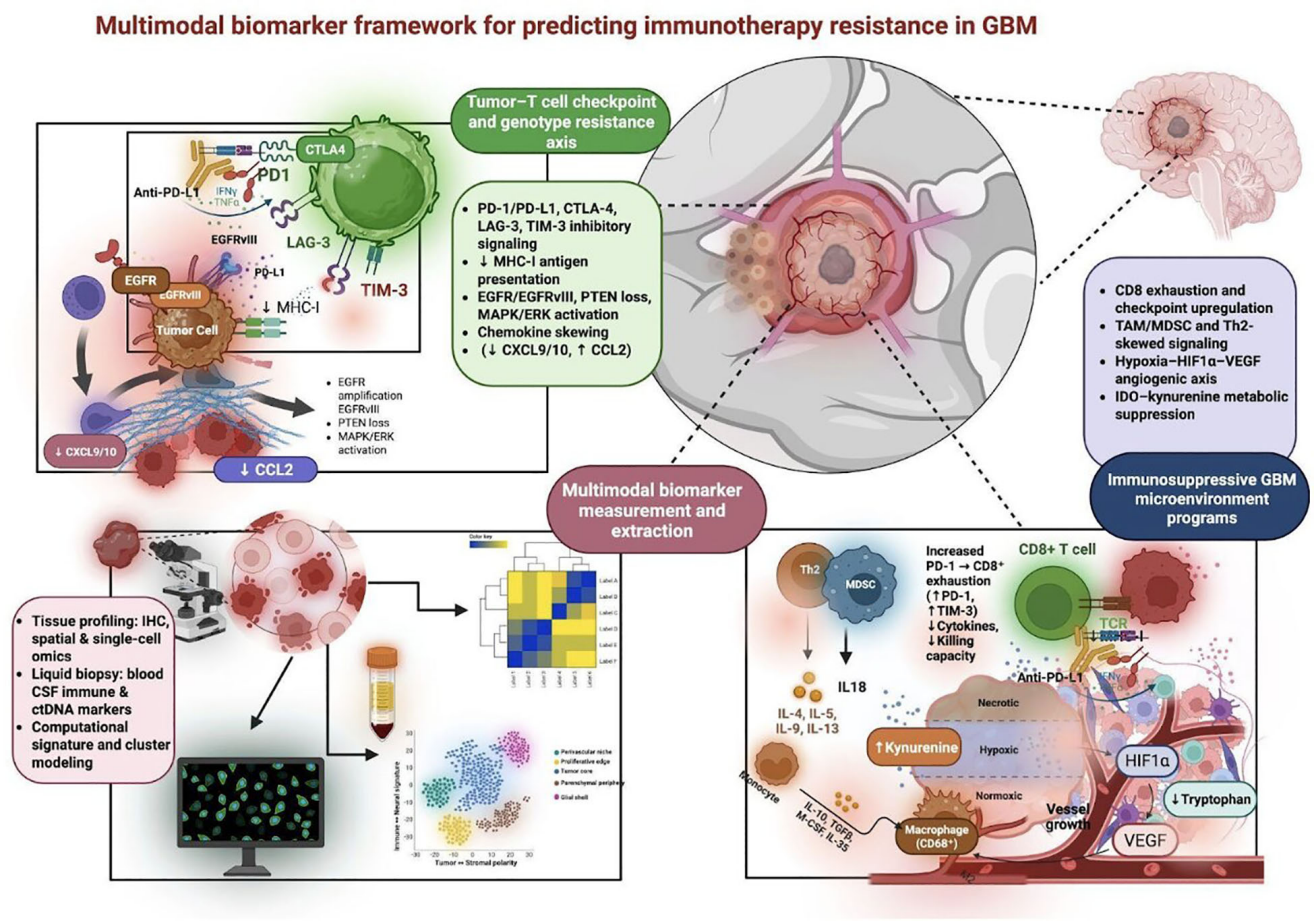
Mechanistic and multimodal biomarker framework for predicting immunotherapy response and resistance in glioblastoma. This schematic illustrates the interactions among tumor intrinsic signaling, immune checkpoint pathways, microenvironmental resistance mechanisms, and multimodal biomarkers, collectively impacting the effectiveness of immunotherapy in GBM. As illustrated at the T cell-tumor cell interface, immune checkpoint pathways (PD-1/PD-L1, CTLA-4, LAG-3, TIM-3), reduced antigen presentation, and genetic mutations (EGFR/EGFRvIII amplification, PTEN deletion, MAPK/ERK activation) contribute to immune evasion and chemokine imbalance ( reduced CXCL9/10 and increased CCL2), which may impact cytotoxic T cell function. The microenvironmental mechanisms that contribute to immune evasion and resistance to immunotherapy in GBM include T cell exhaustion, myeloid cell-dominant immune suppression, hypoxia, HIF-1α-VEGF, and IDO. These mechanisms may be assessed using various multimodal biomarkers as discussed below. The use of these prediction and sequencing models will enable the development of a novel therapy-specific biomarker signature to predict potential responders versus non-responders in longitudinal clinical trials involving immunotherapy in GBM. The figure was made using Biorender (Feb 2026). GBM, glioblastoma; PD-1, programmed cell death protein 1; PD-L1, programmed death-ligand 1; CTLA-4, cytotoxic T-lymphocyte–associated protein 4; LAG-3, lymphocyte activation gene 3; TIM-3, T-cell immunoglobulin and mucin domain–containing protein 3; EGFR, epidermal growth factor receptor; EGFRvIII, epidermal growth factor receptor variant III; PTEN, phosphatase and tensin homolog; MAPK, mitogen-activated protein kinase; ERK, extracellular signal–regulated kinase; CXCL9/10, C-X-C motif chemokine ligand 9/10; CCL2, C-C motif chemokine ligand 2; HIF-1α, hypoxia-inducible factor 1 alpha; VEGF, vascular endothelial growth factor; IDO, indoleamine 2,3-dioxygenase.

The mitogen-activated protein kinase (MAPK) family is heavily involved in pan-cancer oncogenesis, including GBM migration, proliferation, and survival (119) like other tyrosine kinases (120). Indeed, mutations within the MAPK/ERK pathway can be important molecular distinguishers–such as *BRAFV600E* distinction between classic GBM and epithelioid GBM (121–126). Moreover, tumor-intrinsic MAPK/ERK hyperactivation was associated with sensitization of tumors to anti-PD-1 therapy and improved survival in murine models (126). As a biomarker for GBM, mutations of the MAPK pathway–largely *BRAF* and *PTPN11* subcomponents–were significantly enriched in responders to ICB (those with robust immune infiltrate or with imaging-based tumor size stability or reduction) than non-responders in rGBM (OR = 12.8, *p* < 0.05) (24). Downstream effects of the MAPK pathway (as seen in **Figure 1**), studied through phosphorylation of *ERK1/2*, found that increased density of activated or phosphorylated *ERK1/2* was correlated with positive response to ICB therapy in rGBM (*p* < 0.0029) (127) and acted as a biomarker for ICB sensitivity and survival benefit in clinical trials (128). Even so, MAPK pathway mutations are rare in GBM (estimated at 7.8%) (24) and, therefore, may have low utility as biomarkers in GBM.

### Interferon signaling

Type I and II interferon (IFN) signaling pathways are the most commonly implicated in gliomas (25) and GBM (129) among the three IFN signaling families. Mechanistically, Type I IFN signaling agonizes the type I IFN receptor (IFNAR)–composed of IFNAR1 and IFNAR2 subunits–through several cytokine subtypes including IFN-α and IFN-β (130, 131). Induction of Type I IFN signaling is most often for the antiviral response, although induction of the Type I IFN pathway is also seen for bacterial infections through Toll-like receptors (TLR) (132, 133). Type II IFN signaling depends on IFN-γ receptor (IFNGR) activation by IFN-γ, predominantly by natural killer (NK) cells (134, 135). Both signaling pathways are involved in the antiviral immune response (136) but have distinct and context-dependent immunomodulatory roles that can induce pro-apoptotic and anti-proliferative genes (137, 138) and anti-apoptotic genes of the B cell lymphoma (BCL) family oncogenes through the downstream phosphorylation of STAT3 (139, 140). An important determinant of whether IFN signaling exerts antitumoral or protumoral effects is the temporal dynamics of pathway activation, with acute and chronic signaling driving distinct downstream consequences. In the acute setting, Type I IFN signaling activation results in the phosphorylation of STAT1 and STAT2 for the activation of IFN signaling genes (ISG). This short-term cascade results in antitumoral cytotoxicity, commonly in response to exogenous therapeutics such as radiotherapy (141, 142). In contrast, chronic Type I IFN signaling contributes to a protumoral effect and generates resistance to DNA damage through a novel unphosphorylated STAT (U-STAT) pathway that activates IFN-related DNA damage resistance signature (IRDS) genes (143). In cancer cells, high levels of PD-L1, irrespective of environmental immunogenicity, constitutively express IFN-β through the cGAS–STING pathway for the production of IRDS genes (144, 145). IFN-γ plays a similarly dichotomic role with less defined effects between acute and chronic signaling (146).

In GBM, IFN signaling is a highly context-dependent downstream pathway of immune activation (147) implicated in both the antitumoral, immunogenic abscopal effects of immunotherapy and chemoradiation (148), as well as the pro-tumoral upregulation of immunosuppressive signaling and PD-L1 hyperexpression in the tumor microenvironment (149). Regarding the pro-tumoral effects of IFN signaling specific to GBM, ISG are highly expressed in GBM (149) and facilitate immunosuppressive microenvironments through the upregulation of PD-L1 (149–151) and IDO1 (152). High Type I IFN gene expression (*PRDX1, SEC61B, XRCC5, and Troxerutin (TXN)*) was associated with poor survival in lasso-penalized Cox analysis and Kaplan-Meier analysis (*p* < 0.05) in a bioinformatics, exploratory study of the TCGA (153). In regard to the anti-tumoral effects of IFN signaling, IFN-γ plays an important role. Ex vivo drug response platform of GBM explanted tissue demonstrates that histological IFN-γ signaling was higher in anti-PD-1 treated tumors (154, 155). Similarly, high expression of IFN-γ acted as a prognostic and predictive biomarker and was associated with longer OS (p = 0.0015 - 0.038) in pre-treatment newly diagnosed GBM subsequently treated with concurrent atezolizumab, radiation therapy, and temozolomide (156). Another investigation found that a 23 gene-signature of IFN-γ genes derived from bulk RNA-sequencing of rGBM treated with neoadjuvant pembrolizumab was associated with longer OS and PFS (157). IFN-γ signaling is also related to the dose and duration of ICB regardless of survival. In patients with rGBM undergoing both neoadjuvant and adjuvant pembrolizumab therapy versus adjuvant pembrolizumab alone, IFN-γ gene expression signatures were consistently upregulated in neoadjuvant treated rGBM versus adjuvant therapy alone (26, 158). In a recent Phase I/II clinical trial of oncolytic adenovirus (DNX-2401) and pembrolizumab combination therapy, one patient out of ten patients with biopsy at the time of disease progression after treatment had an objective response. In this patient, genes associated with IFN-γ signaling and a related inflamed TME were overexpressed after treatment compared to the non-responding patients (159). With ICB therapy, increased parenchyma expression of IFN-γ genes is a plausible predictive and prognostic biomarker candidate and should be investigated prospectively in controlled trials. Conversely, Type I IFN gene signatures as a predictive biomarker are exploratory but have strong biological plausibility and should be investigated in controlled retrospective studies as an inversely-related predictor of response.

### Epidermal growth factor receptor

Epidermal Growth Factor Receptor (EGFR) mutations are common pan-cancer prognostic biomarkers (160). Preclinical studies suggest that EGFR plays an important role in GBM immune resistance. EGFR alterations have also been associated with increased tumor-associated macrophage (TAM) infiltration into the TME leading to local immunosuppression (161). EGFRvIII mutations specifically have a higher proportion of MDSCs in the TME compared to EGFR wildtype mice (162), as EGFRvIII mutation promotes MDSC migration into tumor parenchyma from the peripheral blood and spleen. Combined anti-PD1/anti-CTLA-4 ICB led to sustained growth arrest in mice with EGFR-wildtype GBM, but not mice with EGFRvIII mutation (163). EGFR mutations, therefore, are thought to play a role in defining the immunogenicity of the TME and have been nominated as both a prognostic and predictive biomarker in GBM immunotherapy. In human GBM, EGFR mutations and amplification occur frequently (160, 164, 165). Of EGFR mutation variants, EGFRvIII is the most common variant (166) and is tumorogenic–promoting proliferation, TMZ resistance, and glycolysis (167, 168). Yet, the roles of EGFR mutations as a general prognostic biomarker for GBM have been controversial despite evidence correlating overexpression with poor survival (169, 170). A recent meta-analysis of 4208 GBM patients continues this trend, demonstrating inconclusive evidence of both EGFR and EGFRvIII amplification in GBM prognostication. Still, EGFR mutations play an important role in stratifying GBM subtypes, largely classical GBM, and acts a potential marker for therapy-specific response (171) such as bevacizumab therapy responsiveness (p=0.01) (172). For ICB therapy, pre-treatment EGFR and EGFRvIII amplification were found to be important markers for response to either pembrolizumab or nivolumab with bevacizumab in a small, exploratory cohort study. Next-generation sequencing (NGS) of 11 tumor samples taken prior to treatment revealed that EGFR gene amplification and EGFRvIII mutation were both associated with worse response to ICB (HR 12.2, 95% CI 1.37–108, p = 0.025). This relationship was also seen in tumor tissue taken at any time point after diagnosis and during treatment (HR 3.92, 95% CI 1.03–14.9, p = 0.045) (173). As less invasive tests continue to be developed to identify EGFR gene amplification or EGFRvIII mutation (174–176), the prognostic and predictive value of EGFR mutations in identifying candidates for ICB and other immunotherapies will be further elucidated. However, there is insufficient evidence at this time for EGFR mutations and the EGFRvIII mutation as predictive or prognostic biomarkers for ICB therapy and further validation in cohort studies is required.

### Circulating cell-free DNA

Circulating cfDNA has been used in advanced solid tumors for detecting tumor burden, monitoring treatment response, or recurrence surveillance (177, 178). As discussed, cfDNA can be detected from a wide-range of biofluids including blood, CSF, and tumor *in-situ* fluids (TISF) (179). Studies for the clinical use of cfDNA for GBM is still limited (180) but recent clinical trials have started to identify TISF-cfDNA as an important biomarker candidate for ICB therapy. In an open-label phase 2 study of bevacizumab and tislelizumab treatment in rGBM, dynamic changes of cfDNA abundance in TISF was significantly correlated with therapy response as defined by the RANO 2.0 criteria. Although baseline TMB detected in cfDNA was not associated with improved prognosis in this study, dynamic decreases in TMB in cfDNA collected was associated with improved overall survival (OS) and PFS (181). In a similar single center, single-arm, open-label, Phase IIa trial of bevacizumab and tislelizumab treatment in rGBM, decreasing levels of cfDNA abundance overtime also predicted improved OS and PFS. Dynamic changes in cfDNA is a strong predictive and prognostic biomarker candidate for GBM immunotherapy and should be thoroughly investigated as a parallel tool to traditional neuroimaging for response monitoring (179). Moreover, the feasibility of safe serial collection of TISF demonstrated by the Phase II clinical trials indicates possible future clinical utility.

## Section 4. Biomarkers of vaccination therapy and virotherapy

### Virotherapy

Oncolytic viral therapy is a promising immunotherapy that leverages oncolytic viruses (OV) to kill tumor cells and stimulate immune activation through the release of neoantigens (28). Although overall response rates across trials have been modest, several studies have demonstrated durable responses in small subsets of patients (182–186). In the phase I PVSRIPO study of recombinant poliovirus, overall survival reached 21% at 24 months (95% CI, 11–33) and remained stable through 36 months, demonstrating a survival plateau consistent with a durable-responder tail within the rGBM population (185). Similarly, a Phase II/III clinical trial of the vocimagene amiretrorepvec vaccine injection (Toca 511) in combination with the oral prodrug 5-flucytosine (Toca FC) in rGBM found that, although there was no improvement in OS in the cohort as a whole, specific subgroups such as *IDH*-mutant HGG in their second recurrence demonstrated increased survival (187). This confirmed preceding Phase I exploratory analysis of the Toca 511/Toca FC trial that associated better prognosis with *IDH*-mutant status (188).

The same Phase I clinical trial also found that enrichment of the B*07-Cw*07 haplotype and tumor neoantigens arising from the *IDH1, PIK3CA, EGFR*, or *SYNE1* genes were associated with prolonged (>2 year) survival in the treatment group, although this was not confirmed in the Phase II/III trial analyzes (188). IFN signaling has also emerged as a potential biomarker for virotherapy efficacy. In a Phase I trial of attenuated measles virus (MV) therapy, ISG expression analysis demonstrated that constitutive IFN activation was correlated with reduced MV virotherapy efficacy in patient-derived xenografts. Indeed, the JAK1/2 inhibitor ruxolitinib was able to attenuate the effects of Type I IFN activation on MV virotherapy resistance in the same study, demonstrating the efficacy of ISG expression as a biomarker for identifying possible candidates for combinatorial therapy (189). That said, there is limited evidence for investigating the *IDH1, PIK3CA, EGFR*, or *SYNE1* genes and B*07-Cw*07 haplotype further as predictive biomarkers of virotherapy. ISG expression, however, should continue to be explored in exploratory preclinical and bioinformatic studies and progress to cohort studies as a strict predictive biomarker due to strong relationship to inherent GBM immunogenicity as discussed in the ICB section.

### Dendritic cell vaccines

Dendritic cell (DC) vaccination was first shown in 1999 to prolong survival in 9L glioma rat models and enhance infiltrating cytotoxic T-cell activity (29). Since then, numerous clinical trials have been initiated to evaluate its efficacy in GBM (29, 30, 190–194) with several exploring biomarkers of immunotherapy responsiveness (31–35, 195–198). Phase I/II trial data and one Phase III clinical trial (30) of autologous DC vaccination immunotherapy in newly diagnosed GBM found that *MGMT* methylation (31, 32, 34, 192, 193, 195, 196) and extent of resection (EOR) were both independent predictors of OS and PFS as noted in **Table 3** (195). Consistent with these predictors, the randomized phase II ICT-107 trial demonstrated that benefit was only seen in subgroups of HLA-A2+/*MGMT*-methylated patients (median PFS was 24.1 months with ICT-107 versus 8.5 months with control (HR 0.26; P = 0.004)) and HLA-A1+/*MGMT*-methylated patients (median OS 47.6 vs 25.8 months; P = 0.049) (194). *MGMT* methylation and EOR are considerable prognostic and predictive biomarker candidates for GBM DC vaccination therapy tested in clinical trials. Even so, both *MGMT* methylation and EOR remain unreliable due to clinical heterogeneity and their interdependence with other components of SOC treatment in GBM (2).

**Table 3:** Current state of biomarkers for vaccine-based immunotherapies.

Identified biomarker (reference)	Source	Correlation to survival (OS/PFS)	Vaccine/viral therapy used	Level of evidence as a predictive biomarker
MGMT Methylation (30)	Tumor	Positive	DC Vaccine/HSPPC-96 Peptide Vaccine	Clinical Trials/Insufficient evidence as predictive biomarker for immunotherapy
Extent of Resection (31)	Tumor	Positive	DC Vaccine	Clinical Trials/A clinical variable; insufficient evidence as Predictive Biomarker
Above Median Peripheral CD 8+ T-cells Count (33)	Peripheral Blood	Positive	DC Vaccine	Clinical Trials/*Post hoc* correlative evidence; insufficient evidence as a validated predictive biomarker
IFN-y Expression (33)	Peripheral Blood	Positive	DC Vaccine	Clinical Trials/Correlative pharmacodynamic and prognostic evidence; growing evidence as a validated predictive biomarker
HLA-A2+ status (32)	Tumor	Positive	DC Vaccine	Clinical Trials/Subgroup enrichment evidence; therapy-specific predictive biomarker candidate
High PD-1+/CD8+ Ratio in Tumor-Infiltrating Lymphocytes (34)	Tumor	Negative	DC Vaccine	Clinical Trials/*Post hoc* correlative evidence; insufficient evidence as a validated predictive biomarker
CD 8+ T cell Responsiveness to IL-2 (199)	Peripheral Blood	Positive	DC Vaccine	Clinical Trials/Correlative evidence; exploratory evidence as a validated predictive biomarker
Low PD-L1 Expression in Myeloid Cells (36)	Peripheral Blood	Positive	HSPPC-96 Peptide Vaccine	Clinical Trials/Phase II correlative evidence; plausible predictive biomarker candidate
Low T-Cell Receptor Diversity (38)	Tumor	Positive	HSPPC-96 Peptide Vaccine	Clinical Trials/Correlative evidence; predictive biomarker candidate requiring further validation
Low MxA expression (39)	Peripheral Blood	Positive	HSPPC-96 Peptide Vaccine	Clinical Trials/Emerging correlative evidence; insufficient evidence but biological plausible predictive biomarker candidate
*SDC-4* Levels in Circulating Tumor DNA (41)	Peripheral Blood	Negative	Wilm’s Tumor 1 Peptide Vaccine	Clinical Trials/Emerging *post hoc* evidence; insufficient evidence as a validated predictive biomarker

This table summarizes clinically evaluated biomarkers associated with response defined by survival time to dendritic cell (DC) vaccines, heat shock protein peptide complex-96 (HSPPC-96) vaccines, and Wilms tumor 1 (WT1) peptide vaccines in glioblastoma (GBM). Biomarkers are categorized by source compartment, direction of association with clinical outcomes, and level of supporting evidence. This table highlights the most generalizable and clinically promising candidates discussed in the text and is not exhaustive.

GBM, glioblastoma; DC, dendritic cell; HSPPC-96, heat shock protein peptide complex-96; WT1, Wilms tumor 1; MGMT, O-6-methylguanine-DNA methyltransferase; PD-1, programmed cell death protein 1; PD-L1, programmed death-ligand 1; IFN-γ, interferon gamma; HLA, human leukocyte antigen; IL-2, interleukin-2; CD8, cluster of differentiation 8; MxA, myxovirus resistance protein A; SDC-4, syndecan-4; ctDNA, circulating tumor DNA; OS, overall survival; PFS, progression-free survival.

Peripheral immune cell quantification has been identified as a potential pre-treatment biomarker of DC vaccination efficacy. *Post-hoc* analysis of Phase II clinical trial data of autologous DC vaccine therapy in newly diagnosed GBM (30) demonstrated that patients with tumor infiltrating lymphocytes with a lower PD-1+/CD8+ ratio had markedly prolonged OS and PFS (196). In *post-hoc* analysis of a Phase II clinical trial utilizing autologous IL-12 secreting dendritic cells activated with autologous tumor lysate, above-median pre-vaccination peripheral CD8+ T-cell and monocyte counts were associated with prolonged OS (p = 0.005) (33), pre-vaccination ELISPOT IFN-γ levels were associated with prolonged OS (p = 0.037) and PFS (p = 0.040), and quantity of circulating T-regulatory (Treg) cells were associated with decreased OS (p = 0.0001) (33). In exploratory analysis comparing two Phase I clinical trials of glioma-associated antigen (GAA) peptide-loaded versus autologous tumor lysate-loaded DC vaccination (35, 198), investigators found that a pre-vaccination/post-vaccination Treg ratio of less than 0.8865 and a pre-vaccination/post-vaccination CTLA-4 expression ratio on CD8+ T-cells of less than 0.8065 were associated with prolonged OS (35). Additionally, increasing peripheral CD 8+ T cell responsiveness to IL-2 and subsequent changes to levels of STAT-5 were associated with long-term survival (199). Collectively, these findings underscore the importance of immunophenotyping in GBM biomarker nomination. However, significant study design variations in immune metrics across clinical trials currently limits its reliability, highlighting the need for standardized approaches to establish robust predictive and prognostic biomarkers in DC vaccination.

Exploratory proteomic and miRNomic analyzes have also been used to understand predictors of DC vaccine response. In a *post-hoc* analysis of the previously mentioned Audencel trial, individual proteins that were associated with poor prognosis included Huntingtin interacting protein 1 (*HIP1*), retinol binding protein 1 (*RBP1*), and chromosome 9 open reading frame 64 (*C9orf64*), and proteins associated with better outcomes included insulin-like growth factor receptor 2 (*IGFR2*). In the MiRNomic analysis, miR-216b, miR-216a, miR-708 and let-7i were identified as possible markers of long-term response (200), however, this was the first study of its kind and miRNA signatures require further validation.

### Peptide vaccines

Peptide vaccines mount an immune response against GBM tumor cells through the presentation of tumor-associated and tumor-specific antigens (TAA and TSA) to cytotoxic T cells. Types of peptide vaccines include those that express global TAAs and TSAs present across large cohorts of patients, as well as personalized peptide vaccines specifically designed for individual patients (201).

Heat shock proteins (HSP) are a family of proteins involved in intracellular chaperoning, including during antigen presentation, and HSP-antigen complexes can be used to trigger antitumor immunity (202). Because of this capability, vaccines developed from purifying HSP-antigen complexes have become increasingly appealing (203), with early tolerability demonstrated in recurrent HGG (204, 205). Heat shock protein peptide complex-96 (HSPPC-96) vaccines have been trialed in newly diagnosed GBM with promising tolerability (36, 37). In a phase II single-arm, multi-centered trial of HSPPC-96 vaccination in combination with standard therapy in newly diagnosed GBM, HSPPC-96 vaccinations demonstrated survival benefits based on historical controls. In addition, the phase II clinical trial demonstrated that *MGMT* methylation status and low PD-L1 expression in peripheral myeloid cells were associated with improved survival with HSPPC-96 therapy (36). A separate Phase I open-label clinical trial of HSPPC-96 vaccination in combination with standard of care, tumor-specific immune response (TSIR) was calculated by stimulating PBMCs with autologous tumor lysate in an IFN-γ release ELISPOT assay. Baseline (pre-vaccine) TSIR did not predict outcome, but post-vaccination TSIR—dichotomized at the cohort median—stratified progression-free survival, with high post-vaccine TSIR associated with improved PFS (HR 0.32; 95% CI 0.11–0.94; P = 0.038) (37). Building on these findings, a study by the same group found that *MGMT* methylation was not significantly associated with long-term survival (38) with insufficient for clinical utility in GBM peptide-based immunotherapy. However, lower T-cell receptor (TCR) Shannon entropy index–a TCR diversity metric that accounts for how many distinct clonotypes of TCR are present (206) was associated with improved OS (38). Indeed, long-term survivors shared high levels of similarity between TCR clones. *CDR3–1* and *CDR3–2* TCR clonotypes predicted survival, demonstrating that clonotypes contributed to vaccine success. Multidimensional immunofluorescence (MIF) was then applied to the aforementioned clinical trials data. In MIF analyzes of immunohistochemical slides, peripheral CD4+ T-cells, CD8+ T-cells and PD-1+ cells densities did not significantly predict survival. Instead, low MxA expression–an IFN-stimulating gene product–differentiates long-term and short-term survivors (AUC = 0.7955, P = 0.0318) independent from TSIR (39). MxA is specifically induced by IFN-α/β and acts as a reflection of the previously discussed Type I IFN-α/β signaling pathway (207–209). As such, the study authors hypothesize further involvement of IFN-pathways in GBM immunogenicity. The predictive and prognostic biomarker candidates for HSPPC-96 vaccination all remain exploratory and divergent. As a result, focused preclinical efforts to confirm candidates related to immunogenicity and IFN signaling should be conducted alongside current RCTs.

Wilm’s tumor 1 (WT1) is also a peptide of interest that has shown early clinical tolerability and efficacy. A Phase II clinical trial of the WT1 vaccine in 21 WT1/HLA-A*2402-positive rGBM shows vaccine tolerability in patients with a median PFS of 20.0 weeks (210). *Post-hoc* analysis of this Phase II clinical trial in rGBM was conducted for predictive and prognostic biomarker discovery. In this study, WT1 expression level was positively associated with OS/PFS (40). In another *post-hoc* analysis, blood circulating DNA (cDNA) was interrogated for possible biomarkers in peripheral blood mononuclear cells (PBMCs). 32 genes were identified on cDNA microarray analysis and were validated by q-PCR. After rigorous survival analysis, only *SDC-4* negatively correlated with OS (HR: 13.8; 95% CI: 1.35–84.2; p = 0.027) (41). Continued investigation of biomarkers for WT1 responsiveness will be essential moving forward.

Personalized peptide vaccines (PPV) are a promising precision immunotherapy strategy where vaccine therapy is tailored to patient-specific peptides and is safe and feasible in GBM based on real world data (211). A Phase I clinical trial study of 25 patients drew from a warehouse of 48 peptides to develop 4 peptide vaccines based on patient preexisting peptide-specific immunoglobulin IgG levels and HLA subtype (212). As vaccines were well tolerated in this Phase I trial, the same group pursued another Phase I study for 4 peptide vaccine therapy in HLA-A24 patients from 14 pre-selected peptides. This study found similar tolerability with patients receiving up to their 79th vaccination (213). As such, Phase III trial was pursued for PPV in the HLA-restricted rGBM patient cohort. Unfortunately, the study did not meet their primary endpoint and found that PPV did not improve median OS over placebo. However, the study team found that several blood-based predictive and prognostic biomarkers related to PPV responsiveness. Lower immunosuppressive monocytes (p=0.027), higher CD3+CD4+CD45RA− T cells (p=0.031), and intermediate levels of CCL2 related to prolonged OS in their study in those who responded to PPV therapy. In contrast, very high baseline GM-CSF was associated with poorer survival in PPV-treated patients (214).

## Section 5. Radiographic biomarkers of immunotherapy response

Radiographic biomarkers have always been important for the diagnosis and treatment response assessment in GBM, with the utility of radiographic biomarkers only increasing with the use of machine learning and artificial intelligence (AI) (215). In GBM, MRI is routinely used for the assessment of response to therapy through a battery of structural and physiological MRI modalities (216) and is the gold-standard for identifying responsiveness as previously discussed. As such, MRI is invariably the imaging of choice for radiographic biomarkers of GBM. Diffuse MRI-derived Apparent Diffusion Coefficient (ADC) values in a retrospective cohort of 44 IDH–wild-type rGBM patients treated with pembrolizumab or nivolumab demonstrates correlation between imaging features and survival. Using Kaplan–Meier stratification, higher post-ICB median relative ADC (rADC) within enhancing tumor predicted longer survival (10.3 vs 6.1 months for rADC ≥1.63 vs <1.63; P = .02; HR = 0.41), while tumor volume, pre-ICB rADC, and changes in rADC/volume were not significantly associated with OS. In Cox modeling, post-ICB rADC remained independently associated with OS after adjusting for age/sex and even after additionally adjusting for post-ICB debulking surgery (HR: 0.29; P = 0.045) (44). Beyond survival, an exploratory study of 21 patients found that MR susceptibility imaging–a MRI technique useful for identifying locoregional inhomogeneity in tissue including differences in iron and blood degradation products (217)–correlates with L-ferritin positivity percent in histological staining through quantitative susceptibility mapping (QSM)-based mean susceptibility measurements. The higher QSM-based mean susceptibility is co-correlated with CD68 and CD86 stain enrichment. As such, the authors note that future MRI detection of iron can be used to differentiate M2-phenotype TAM versus M1-phenotype TAM infiltration in the GBM TME (218). This is clinically correlative as M2-phenotype TAM suppress innate immune activity while promoting iron release into the tumor microenvironment while M1-phenotype TAM create an anti-tumor, immunogenic environment and sequester intracellular iron (219, 220). The study demonstrates that radiographic biomarkers can be predictive biomarkers of immunotherapy responsiveness by capturing inapparent elements GBM TME and a prognostic biomarker through correlations with OS. Despite the high feasibility of integrating novel neuroimaging findings as GBM predictive and prognostic biomarkers in current workflows, rigorous and standardized investigations in clinical trials have not been completed.

Radiogenomics is the study of unifying genomic and imaging phenotypes (221, 222), and several studies to-date have demonstrated radiogenomic biomarkers for immunotherapy response in GBM (223). For example, MRI-derived ADC and normalized relative cerebral blood volume (nCBV) correlated with tumor RNA expression of immune marker genes including markers of T cells (CD3E), bone marrow–derived myeloid cells (CD49D/ITGA4), and TAM/MDSC-related markers (CD68, CSF1R, CD33) (45). These myeloid populations comprised both infiltrating monocyte-derived macrophages and resident microglia, which represent transcriptionally and spatially distinct compartments within the GBM tumor microenvironment (224). TAM and MDSC can reshape macroscopic features of GBM through tumor angiogenesis and growth that can be detected by MRI tools such as nCBV (225, 226). A similar study leveraged ADC values and relative cerebral blood volume (rCBV) to classify the TME based on T-cell fraction (enriched vs deficient), T-cell subclass fraction (CD8 T-cell vs CD4 T-cell dominant), and M2-phenotype TAM infiltration fraction (M2-phenotype TAM high vs low) (227). This study similarly bridges imaging findings with physiological effects of specific immune populations in the GBM parenchyma. Another study using publicly available data correlated six imaging features (e.g. kurtosis, contrast, small zone size emphasis, low gray level zone size emphasis, high gray level zone size emphasis, small zone high gray level em]phasis) from pre-surgical T1-weighted post-contrast and T2-weighted Fluid-Attenuated-Inversion-Recovery (FLAIR) MRI scans with CD3^+^ cell infiltration with an accuracy of 76.5% and area under the curve (AUC) of 0.847 (228). Although untested in RCTs, radiogenomics provides the opportunity for a highly feasible and safe predictive biomarker across GBM timepoints that bridges several biomarker compartments discussed previously.

## Section 6. Biomarkers identified in ongoing clinical trials

Emphasis on biomarker discovery and implementation in clinical trials is paramount for the reduction risk and participant attrition in clinical trials through early screening and drug delivery prioritization (229). As noted in another review, clinical trials play an especially important role for identifying biomarkers for immunotherapy responsiveness in GBM (46). This study sought to thematically interpret trends in biomarker identification and utilization practices in ongoing clinical trials in GBM immunotherapy. In this review, an informal, targeted search of Clinicaltrials.gov on January 04, 2026 using the following terms: “glioblastoma,” “immunotherapy,” “vaccine therapy,” “CAR T-cells,” and “oncolytic virus.” Trials were screened based on study descriptions and only studies with protocols or endpoints containing clear language and intent for biomarker identification were included. Trials that were completed, terminated, or lacking any mention of biomarker-related endpoints were excluded. Duplicate or overlapping trials were identified by NCT number and consolidated into a single entry during screening. Only trials that were active or recruiting were included to capture the current state of ongoing clinical trials and biomarker identification efforts. Even so, as descriptions of clinical trials can often contain aspirational language, the following discussion acknowledges that intended biomarker discovery does not equate to biomarker validation or analysis. Of the 96 total queried ongoing trials, 36 were included in the final analysis for direct language of biomarker identification efforts in the study descriptions (**Table 3**). Study protocols are thematically analyzed into sample compartments (e.g. cerebrospinal fluid, blood/plasma, tumor parenchyma), cell-based biomarkers (e.g. peripheral immune cell population counts, intratumoral cell population counts, immune cell phenotypes), radiographic biomarkers, cytokine biomarkers including IFN signaling quantification, protein/molecular biomarkers, genetic biomarkers (e.g. circulating tumor DNA, transcriptomics, bulk RNA sequencing), and “other” biomarkers such as delayed-type hypersensitivity skin test 24 hours after therapy. One study (NCT02455557) did not have clear descriptions of analytes included in the study protocol but still fit the inclusion criteria and was maintained as such with “Unknown” in the described columns.

Among the 36 ongoing clinical trials, the majority (n = 29) include biomarker-based endpoints investigating immune cell populations. These assessments most commonly involve longitudinal peripheral blood immunophenotyping using flow cytometry, ELISpot-based IFN-γ secretion assays, and cytokine profiling, with a smaller subset incorporating immunohistochemistry, immunofluorescence, or TCR sequencing. IFN-γ is the predominant cytokine in biomarker discovery as a measure of post-therapy immunogenicity most often in relation to peripheral T-cell responsiveness (NCT04808245, NCT04968366). Radiographic biomarkers are incorporated less frequently (n = 7), but their presence reflects a growing interest in imaging-based correlates of immune response. These studies employ advanced MRI parameters—including diffusion-weighted imaging and perfusion sequences—as well as immuno–positron emission tomography (PET) approaches for dynamic quantification of tumor immune activity (NCT05235737). Notably, although a wide array of exploratory endpoints are included across studies, contemporary trial designs reflect a clear shift away from conventional single biomarkers (e.g., PD-1/PD-L1 expression, tumor mutational burden, and MGMT methylation) toward multimodal, signature-based frameworks that integrate immune, molecular, and radiographic features.

When stratified by intervention class, vaccine-based therapies constituted the largest proportion of included trials, followed by ICB, CAR T-cell therapies, and oncolytic viral approaches. Vaccine therapies most frequently incorporate comprehensive immune monitoring endpoints that balance immunogenicity between cell population studies and cytokine and genetic profiling. Moreover, CAR T-cell therapies are an emerging field with promising results reflected in ongoing Phase I/II clinical trials. Still, considerable ethical and scientific advancements must be made to advance CAR T-cell therapies to Phase III clinical trials (89, 230). In this review, principal biomarkers of interest currently are the pervasiveness of CAR T-cells in tumor cyst fluid, peripheral blood, and CSF, longitudinal cytokine profiling, and expression of the target antigen as seen in previous studies of EGFRvIII (43). These biomarker strategies reflect the mechanistic priorities of each platform—vaccines prioritizing immunogenicity amplification and CAR T-cell therapies prioritizing cellular durability.

## Section 7. Predicted molecular biomarkers of tumor immune microenvironment and immunotherapy response

The impact of public data and open data sharing has pronounced impacts on academics and accelerating research (231). These benefits are even more pronounced in the data sharing of clinical trials data (232) and the foreseeable impacts on improving therapeutic lines in GBM. Several studies leveraging publicly available data sources are published in glioma and GBM for exploratory nomination of biomarkers *in silico*. Savage et al. describes several biomarkers discovered *in silico* (e.g. *PTEN* mutations, ERL 1/2 phosphorylation, 4-chemokine signature, FCER1G expression) through a targeted scope including very promising biomarkers that build on previously reported, physiologically-driven biomarkers (46). Consistent with the framework described by Savage et al., candidate biomarkers in this review were included only if they were (1) GBM-specific (2), physiologically reasonable, and (3) supported by secondary analyzes linking molecular features to preclinical or clinical immunotherapy response datasets. Studies exploring exclusively prognostic biomarkers were excluded from the following discussion. The purpose of this section is to demonstrate the rationale for critically appraising and nominating weak evidence, exploratory biomarkers as mentioned before (65) in light of expanding use of publicly available data (161). However, each study described is only computational correlative currently and does not have strong external validation or clinical utility. Previously established biomarkers were not re-reviewed except for *PTEN* alterations, which were retained given their high prevalence in GBM (30–40%) and consistent association with immunotherapy resistance (27, 233, 234). The final list of included biomarkers have secondary analysis that utilizes datasets of preclinical or clinical responsiveness (e.g. in-house histopathology, TISMO database (235), and clinical trials datasets) to immunotherapy rather than simply mechanistic studies of infiltrating cell populations or inflammatory gene signatures. Accordingly, the biomarkers discussed below represent exploratory but GBM-specific molecular candidates with preliminary linkage to immunotherapy responsiveness.

### Early and novel biomarkers of interest

As seen in **Table 4**, several genes and proteins of interest have been identified in these publicly available databases as potential biomarkers of immunotherapy response. Unsurprisingly, most studies interrogated biomarkers of ICB responsiveness rather than other immunotherapy types. Quantification of protein levels can also serve as biomarkers for immunotherapy response. As seen in **Table 5**, Kindlin-3, *TSPAN7*, *NKG2C*, Klotho, and *PTEN* expression and mutations are all candidate biomarkers. Utilizing the Tumor Immune Syngeneic MOuse (TISMO) database (234), high expression of *FERMT3*, encoding Kindlin-3, was correlated with anti-PD-1 responsiveness in murine models (236). The exact mechanism of this correlation is still to be elucidated but is related to the role of *FERMT3* in the regulation of integrin activation–the absence of which results in leukocyte adhesion deficiency type III (237, 238). Low *TSPAN7* gene expression was associated with better prognosis after immunotherapy reception. Indeed, the best prognosis in this subanalysis of clinical data found that low *TSPAN7* and high *PD-L1* expression groups (239). Although the exact mechanism is unknown, *TSPAN7* is associated with several psychiatric conditions (240) and is related to the development of proper neurite spines (241). *TSPAN7*, as such, may have a role in modulating ICB responsiveness through changes in the neuro-immune axis. *Klotho* expression has been shown to correlate with improved DC vaccine efficacy in preclinical models (241). In human samples from the DENDR1 (NCT04801147) clinical trial, high serum *Klotho* expression was associated with both improved PFS and OS. It is hypothesized that *Klotho* expression is correlated with pro-inflammatory macrophages in the TME (241). *PTEN* alterations were also found to be significantly enriched in non-responders compared with responders of anti-PD-1 therapy in rGBM (P = 0.0063) and were associated with immunosuppressive expression signatures (24). As similar trends have been noted in melanomas and uterine leiomyosarcoma (242, 243), the function of *PTEN* as a biomarker and target remain promising and of interest in future studies.

**Table 4:** Biomarker outcomes from ongoing trials in glioblastoma.

NCT number	Study title	Study status	Phases	Intervention	Biomarker modality	Samples studied	Cell-based biomarker	Radiographic biomarker	Cytokine biomarker	Protein/molecular biomarker	Genetic biomarker	Other biomarker
NCT02208362	Genetically Modified T-cells in Treating Patients With Recurrent or Refractory Malignant Glioma	Active but not recruiting	Phase I	Adoptive Cell Immunotherapy	Immune cell profiling + cytokine analysis + tumor antigen expression	Tumor Cyst Fluid, Peripheral Blood, Cerebrospinal fluid, Histology	Yes	None	None	Yes	None	None
NCT03170141	Immunogene-modified T (IgT) Cells Against Glioblastoma Multiforme	Invitation only	Phase I	Adoptive Cell Immunotherapy	Peripheral blood immune checkpoint antibody quantification	Peripheral Blood	None	None	None	Yes	None	None
NCT03389230	Memory-Enriched T Cells in Treating Patients With Recurrent or Refractory Grade III-IV Glioma	Active but not recruiting	Phase I	Adoptive Cell Immunotherapy	Cytokine profiling and tumor antigen (HER2) expression	Tumor Cyst Fluid, Peripheral Blood, Cerebrospinal fluid, Histology	None	None	Yes	Yes	None	None
NCT03491683	INO-5401 and INO-9012 Delivered by Electroporation (EP) in Combination With Cemiplimab (REGN2810) in Newly-Diagnosed Glioblastoma (GBM)	Active but not recruiting	Phase I/II	Adoptive Cell Immunotherapy	Cellular, TCR repertoire, and antigen-specific humoral immune profiling	Peripheral Blood	Yes	None	None	Yes	None	None
NCT04485949	A Phase 2b Clinical Study With a Combination Immunotherapy in Newly Diagnosed Patients With Glioblastoma	Active but not recruiting	Phase II	Adoptive Cell Immunotherapy	Clinical safety laboratory and vital sign assessments	*Unspecified*	Yes	None	Yes	None	None	None
NCT05685004	Study of Neoantigen-specific Adoptive T Cell Therapy for Newly Diagnosed MGMT Negative Glioblastoma Multiforme (GBM)	Active but not recruiting	Phase II/III	Adoptive Cell Immunotherapy	Post-vaccination delayed-type hypersensitivity (24-hour) + early genetic and immunologic immune correlates	*Unspecified*	None	None	None	None	Yes	Yes
NCT04003649	IL13Ra2-CAR T Cells With or Without Nivolumab and Ipilimumab in Treating Patients With GBM	Active recruitment	Phase I	CAR T-Cell Immunotherapy	Cytokine profiling + tumor antigen/PD-L1 expression	Tumor Cyst Fluid, Peripheral Blood, Cerebrospinal fluid, Histology	Yes	None	Yes	Yes	None	None
NCT04214392	Chimeric Antigen Receptor (CAR) T Cells With a Chlorotoxin Tumor-Targeting Domain for the Treatment of MMP2+ Recurrent or Progressive Glioblastoma	Active but not recruiting	Phase I	CAR T-Cell Immunotherapy	T-cell phenotyping and cytokine profiling + chlorotoxin-target antigen expression	Tumor Cyst Fluid, Peripheral Blood, Cerebrospinal fluid, Histology	Yes	None	None	Yes	None	None
NCT04661384	Brain Tumor-Specific Immune Cells (IL13Ralpha2-CAR T Cells) for the Treatment of Leptomeningeal Glioblastoma, Ependymoma, or Medulloblastoma	Active but not recruiting	Phase I	CAR T-Cell Immunotherapy	Target antigen (IL13Rα2) expression + cytokine profiling	Tumor Cyst Fluid, Peripheral Blood, Cerebrospinal fluid, Histology	None	None	Yes	Yes	None	None
NCT04323046	Immunotherapy Before and After Surgery for Treatment of Recurrent or Progressive High Grade Glioma in Children and Young Adults	Active recruitment	Phase I	Immune Checkpoint Blockade	Tumor transcriptomic and immune microenvironment profiling (IFN-γ signature, TIL density/clonality, PD-1/PD-L1) + tumor mutational burden + peripheral T-cell response + advanced MRI–immune correlation	Tumor, Peripheral Blood, Histology	Yes	Yes	None	Yes	Yes	None
NCT04656535	AB154 Combined With AB122 for Recurrent Glioblastoma	Active but not recruiting	Phase I	Immune Checkpoint Blockade	Tumor and peripheral blood single-cell RNA sequencing + spatial Treg/CD8 immune ratio quantification (immunofluorescence)	Tumor, Peripheral Blood	Yes	None	None	None	Yes	None
NCT04977375	Trial of Anti-PD-1 Immunotherapy and Stereotactic Radiation in Patients With Recurrent Glioblastoma	Active recruitment	Phase I/II	Immune Checkpoint Blockade	Tumor TIL density and TCR clonality + CD8 T-cell activation profiling with serial peripheral blood immune assessment	Tumor, Peripheral Blood	Yes	None	None	None	None	None
NCT05235737	The Assessment of Immune Response in Newly Diagnosed Glioblastoma Patients Treated With Pembrolizumab	Active recruitment	Phase IV	Immune Checkpoint Blockade	Immuno-PET imaging (^89^Zr-DFO-Atezolizumab) for quantitative PD-L1 expression and T-cell level assessment	Tumor	Yes	Yes	None	Yes	None	None
NCT06816927	Trial of Glioblastoma Immunotherapy Advancement With Nivolumab and Relatlimab	Not yet recruiting	Phase II	Immune Checkpoint Blockade	Pre/post-treatment change in tumor-infiltrating lymphocytes (FFPE tumor tissue)	Histology	Yes	None	None	None	None	None
NCT03688178	DC Migration Study to Evaluate TReg Depletion In GBM Patients With and Without Varlilumab	Active but not recruiting	Phase II	Novel Immunotherapy	Immune subset dynamics (Tregs) + serum chemokine profiling (CCL3)	Peripheral Blood	Yes	None	Yes	None	None	None
NCT05864534	Phase 2a Immune Modulation With Ultrasound for Newly Diagnosed Glioblastoma	Active recruitment	Phase II	Novel Immunotherapy	MAPK pathway activity (tumor p-ERK) + blood ctDNA	Tumor, Peripheral Blood	None	None	None	Yes	Yes	None
NCT03152318	A Study of the Treatment of Recurrent Malignant Glioma With rQNestin34.5v.2	Active recruitment	Phase I	Oncolytic Virotherapy	Perfusion MRI biomarkers	None	None	Yes	None	None	None	None
NCT03657576	Trial of C134 in Patients With Recurrent GBM	Active but not recruiting	Phase I	Oncolytic Virotherapy	Immune subset phenotyping + intracellular interferon analysis (FACS)	Peripheral Blood	Yes	None	Yes	None	None	None
NCT05139056	Multiple Intracerebral Doses of Neural Stem Cell-Based Virotherapy (NSC-CRAd-S-pk7) for the Treatment of Recurrent High-Grade Gliomas	Active recruitment	Phase I	Oncolytic Virotherapy	Survivin IHC (pre/post) + spatial tumor immune profiling (Vectra) + CSF multiplex immunoassays + RNA-seq (tumor, blood, CSF)	Tumor, Peripheral Blood, Cerebrospinal fluid, Histology	Yes	Yes	Yes	Yes	Yes	None
NCT02455557	SurVaxM Vaccine Therapy and Temozolomide in Treating Patients With Newly Diagnosed Glioblastoma	Active but not recruiting	Phase II	Vaccine Immunotherapy	Antigen-specific immune response profiling	*Unspecified*	*Unknown*	*Unknown*	*Unknown*	*Unknown*	*Unknown*	*Unknown*
NCT02649582	Adjuvant Dendritic Cell-immunotherapy Plus Temozolomide in Glioblastoma Patients	Active but not recruiting	Phase I/II	Vaccine Immunotherapy	Peripheral blood immune phenotyping + antigen-specific responses	Peripheral Blood	Yes	None	Yes	Yes	None	None
NCT03382977	Study to Evaluate Safety, Tolerability, and Optimal Dose of Candidate GBM Vaccine VBI-1901 in Recurrent GBM Subjects	Active recruitment	Phase I/II	Vaccine Immunotherapy	Antigen-specific humoral and cellular immune response profiling (ELISA/ELISPOT)	Peripheral Blood	Yes	None	Yes	Yes	None	None
NCT03548571	Dendritic Cell Immunotherapy Against Cancer Stem Cells in Glioblastoma Patients Receiving Standard Therapy	Active recruitment	Phase II/III	Vaccine Immunotherapy	Delayed-type hypersensitivity and lymphocyte clonal profiling	Tumor	Yes	None	None	None	None	Yes
NCT04015700	Neoantigen-based Personalized DNA Vaccine in Patients With Newly Diagnosed, Unmethylated Glioblastoma	Active but not recruiting	Phase I	Vaccine Immunotherapy	Flow cytometry immune subsets + TCR-seq diversity/clonality and inferred antigen specificity	Tumor	Yes	None	None	Yes	None	None
NCT04201873	Pembrolizumab and a Vaccine (ATL-DC) for the Treatment of Surgically Accessible Recurrent Glioblastoma	Active but not recruiting	Phase I	Vaccine Immunotherapy	Tumor immune microenvironment profiling (IHC: PD-1/PD-L1, CD3/CD4/CD8, Iba-1, Ki-67) + tumor/blood TCR clonality + transcriptomic (RNA-seq/NanoString) and somatic mutation analysis + peripheral T-cell phenotyping	Tumor, Peripheral Blood, Histology	Yes	None	None	Yes	Yes	None
NCT04523688	Vaccination With Autologous Dendritic Cells Loaded With Autologous Tumor Homogenate in Glioblastoma	Active recruitment	Phase II	Vaccine Immunotherapy	Delayed-type hypersensitivity + HLA class I/II typing, tumor antigen expression + peripheral and tumor immune-cell profiling	Tumor, Peripheral Blood	Yes	None	None	Yes	None	Yes
NCT04801147	Immunotherapy With Autologous Tumor Lysate-Loaded Dendritic Cells In Patients With Newly Diagnosed Glioblastoma Multiforme	Active recruitment	Phase I/II	Vaccine Immunotherapy	Longitudinal immune response monitoring during and after vaccination	Tumor	Yes	None	Yes	None	None	None
NCT04808245	A MultIceNTER Phase I Peptide VaCcine Trial for the Treatment of H3-Mutated Gliomas	Active but not recruiting	Phase I	Vaccine Immunotherapy	T-cell immunogenicity (PBMC IFN-γ ELISpot)	Peripheral Blood	Yes	None	Yes	None	None	None
NCT04968366	Safety & Efficacy of DC Vaccine and TMZ for the Treatment of Newly-diagnosed Glioblastoma After Surgery	Active but not recruiting	Phase I	Vaccine Immunotherapy	Tumor-specific T-cell immunogenicity (serial PBMC IFN-γ ELISpot)	Peripheral Blood	Yes	None	Yes	None	None	None
NCT05163080	SurVaxM Plus Adjuvant Temozolomide for Newly Diagnosed Glioblastoma (SURVIVE)	Active but not recruiting	Phase II	Vaccine Immunotherapy	Imaging predictors + molecular predictors of SurVaxM response: MGMT methylation, tumor survivin expression, anti-survivin immunoglobulin titers, and CD8+ T-cell responses.	Tumor	Yes	Yes	Yes	Yes	None	None
NCT05283109	ETAPA I: Peptide-based Tumor Associated Antigen Vaccine in GBM	Active but not recruiting	Phase I	Vaccine Immunotherapy	Antigen-specific T-cell expansion (pp65, EphA2, survivin)	Tumor	Yes	None	None	Yes	None	None
NCT05698199	Study to Evaluate the Safety, Tolerability, Immunogenicity and Preliminary Efficacy of ITI-1001 In Patients With Newly Diagnosed Glioblastoma (GBM)	Active but not recruiting	Phase I	Vaccine Immunotherapy	Peripheral immune activation (ELISpot/flow/CMV serology) + MRI response analysis + tumor IHC (CMV proteins and CD8/CD163/FOXP3) + NANO scale assessment	Tumor, Peripheral Blood, Histology	Yes	Yes	Yes	Yes	None	None
NCT05743595	Neoantigen-based Personalized DNA Vaccine With Retifanlimab PD-1 Blockade Therapy in Patients With Newly Diagnosed, Unmethylated Glioblastoma	Active recruitment	Phase I	Vaccine Immunotherapy	Neoantigen-specific CD8+ T-cell immunogenicity (cohort-level response rate) + immune subset profiling (T-cell phenotype, MDSCs) + TCR-seq clonality/diversity + plasma cytokine/chemokine multiplex ELISA	Tumor	Yes	None	Yes	Yes	None	None
NCT06043232	MMR/MSI Phenotypes in Prediction of Tumor Vaccine Benefit for Gliomas	Active recruitment	Not Applicable	Vaccine Immunotherapy	Transcriptomic (RNA-seq) + TCR/BCR clonality sequencing+ WGS/WES mutation profiling + computational imaging feature analysis + IHC-based protein expression	Tumor	Yes	Yes	Yes	Yes	Yes	None
NCT06132438	Immunotherapy Targeting of Cytomegalovirus Antigens in Glioblastoma	Not yet recruiting	Phase I	Vaccine Immunotherapy	Peak IFN-γ–secreting T-cell response (ELISpot)	Tumor	Yes	None	Yes	None	None	None
NCT06749925	Clinical Trial Assessing the Efficacy and Safety of Dendritic Cell-Based Immunotherapy for Glioblastoma	Not yet recruiting	Phase II/III	Vaccine Immunotherapy	Peripheral Th1-pattern tumor-reactive T lymphocyte levels	Peripheral Blood	Yes	None	None	None	None	None

Table summarizes biomarker-related endpoints explicitly listed in trial records for ongoing glioblastoma immunotherapy studies. Trials were identified by targeted keyword search of ClinicalTrials.gov and included if listed as active at the time of data extraction; biomarker measures were abstracted verbatim and fit into standardized biomarker categories (e.g. immune cell populations/phenotypes, cytokines, imaging, and molecular assays). Outcomes reflect planned or reported biomarker assessments in registry entries.

ADC, apparent diffusion coefficient; AUC, area under the curve; CAR, chimeric antigen receptor; CEST, chemical exchange saturation transfer; CSF, cerebrospinal fluid; ctDNA, circulating tumor DNA; DC, dendritic cell; DFO, desferrioxamine; DWI, diffusion-weighted imaging; ELISA, enzyme-linked immunosorbent assay; ELISpot, enzyme-linked immunospot; FFPE, formalin-fixed paraffin-embedded; FLAIR, fluid-attenuated inversion recovery; ICB, immune checkpoint blockade; IFN-γ, interferon gamma; IHC, immunohistochemistry; IL, interleukin; IP-10, interferon gamma–induced protein 10 (CXCL10); MGMT, O6-methylguanine-DNA methyltransferase; MDSC, myeloid-derived suppressor cell; MRI, magnetic resonance imaging; NANO, Neurologic Assessment in Neuro-Oncology; NCT, ClinicalTrials.gov identifier; PBMC, peripheral blood mononuclear cell; PD-1, programmed cell death protein 1; PD-L1, programmed death-ligand 1; PET, positron emission tomography; rCBV, relative cerebral blood volume; RNA-seq, RNA sequencing; scRNA-seq, single-cell RNA sequencing; SRT, stereotactic radiotherapy; TCR, T-cell receptor; TIL, tumor-infiltrating lymphocyte; TMZ, temozolomide.

**Table 5:** Early and novel biomarkers for immunotherapy response.

Biomarker (reference)	Immunotherapy agent	Correlation with overall survival	Pre-/post- treatment	Level of evidence	Primary datasets used
Kindlin-3 (236)	Anti-PD-1	Positive	Pre-Treatment	Bioinformatic, Clinical	Clinical Proteomic Tumor Analysis Consortium database, Chinese Glioma Genome Atlas, GlioVis
*TSPAN7* gene (239)	Anti-PD-1	Negative	Pre-Treatment	Bioinformatic, Clinical, Preclinical	Chinese Glioma Genome Atlas, The Cancer Genome Atlas, SRA database
*Klotho* *(*242)	DC Vaccination	Positive (in patients with complete resection only)	Pre-Treatment	Clinical, Preclinical	Study Data, Clinical Trial Data
*PTEN* Mutations (24)	Anti-PD-1	Negative	Pre-Treatment	Clinical	Study Data

This table summarizes exploratory biomarkers identified through bioinformatic, preclinical, and early clinical analyzes linking molecular features to immunotherapy responsiveness in GBM as an exercise for nominating biomarkers identified in bioinformatic studies. Biomarkers were included only if supported by secondary analyzes associating molecular expression or mutation status with preclinical or clinical response datasets.

GBM, glioblastoma; DC, dendritic cell; PD-1, programmed cell death protein 1; PTEN, phosphatase and tensin homolog.

### Advanced computational strategies for biomarker identification

Advanced computation strategies have opened the door to novel biomarker discovery and the integration of multi-omic data, ranging from radiomic to molecular data (**Table 1**), that allows for more nuanced precision medicine (244). Various studies have found integrated immunogenic signatures that generate prediction of the GBM TME (245–260). In doing so, machine learning techniques have provided hypothesis-generating correlations in GBM oncogenesis pathways (252), immunotherapy resistance (261), and peripheral biomarkers (262). These studies are not justifiably translational in the current state and lack definitive biological plausibility, external validation, and clinical utility; however, the bioinformatic studies listed advance predictive biomarker discovery and demonstrate the importance of integrated signatures as biomarkers over single biomarkers as done historically.

The use of external data for clinical trials in neuro-oncology is of significant interest (263). As described, publicly available glioma datasets and analysis tools support rapid and reproducible *in silico* biomarker nomination for GBM immunotherapy. Key resources for correlative computational studies for hypothesis-generating biomarker discovery include The Cancer Genome Atlas (TCGA) (264) and Chinese Glioma Genome Atlas (CGGA) (265) for tumor genomics/transcriptomics, Gene Expression Omnibus (GEO) (292) for independent expression datasets, and web-based platforms such as GlioVis (266), CIBERSORT (267), and TIMER2.0 for TME profiling (268). Although beneficial for nominating biomarkers rapidly, these datasets often lack external significance and are limited by heterogeneity in data acquisition, processing pipelines, and patient populations. Datasets such as the Glioblastoma External (GBM-X) Data Platform pools the data of 1,200 individual patients across several controlled trials for study design and external validation to address this gap (263). Datasets like GBM-X are powerful tools for designing more robust, well-powered clinical trials through an externally augmented clinical trial (EACT) design process (269, 270). Any large dataset for GBM immunotherapy biomarker discovery should include harmonized individual level data from previous controlled trials with endpoints that benefit future study design. Similarly, algorithms and AI computation for biomarker discovery and clinical trials must be adequately reported (271, 272, 293). Algorithms should have standardized endpoints and outcomes that better serve future clinical trials designs. For predictive biomarkers in GBM immunotherapy, an optimal training endpoint has not been established by international consensus; however, clinical trials most commonly define treatment response using RANO 2.0 criteria or OS/PFS.

### Future directions

**Figure 2:**
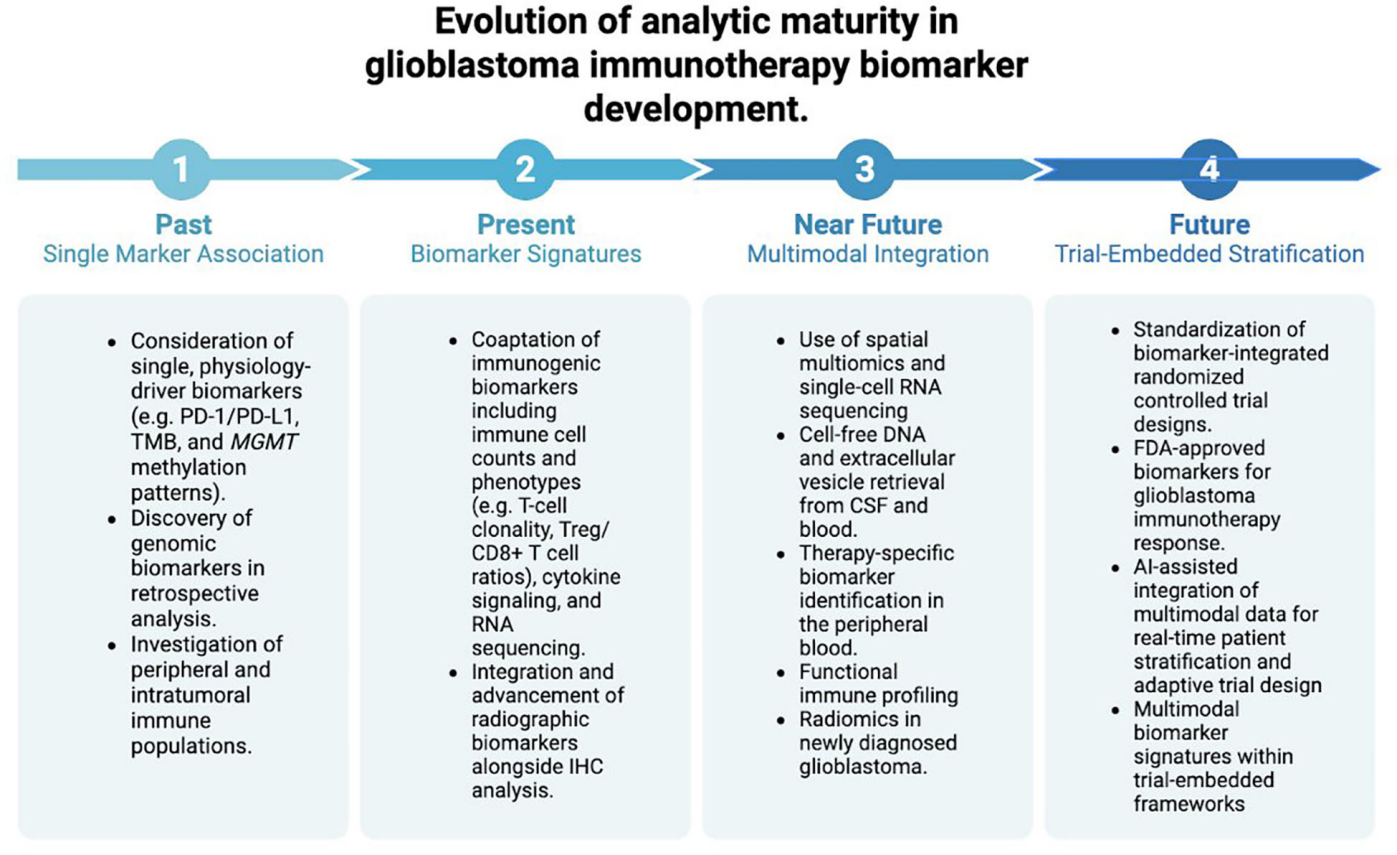
Evolution of analytic maturity in glioblastoma immunotherapy biomarker development. This schematic illustrates the maturation of biomarker development strategies in GBM immunotherapy. Past endeavors include single biomarker discovery and testing. Present efforts include multimodal signature discovery and advancements in integrating radiographic features in immunogenic biomarkers. Near future efforts in biomarker discovery include further integration of liquid biopsies in clinical trials. Future use of biomarkers may include standardization of controlled trial frameworks for embedded biomarker discovery. GBM, glioblastoma; PD-1, programmed cell death protein 1; PD-L1, programmed death-ligand 1; TMB, tumor mutational burden; MGMT, O-6-methylguanine-DNA methyltransferase; CSF, cerebrospinal fluid.

The next phase of biomarker utilization in GBM relies on uniformity of validated, therapy-specific frameworks embedded within immunotherapy trials that consider emerging technologies and AI (**Figure 2**) (273). Biomarkers must move from retrospective, single-marker discovery towards multimodal signatures of analytes from several different compartments. Advancements in sonobiopsy (274–276), although early and only proof-of-concept, exemplify innovative approaches towards expanding biomarker retrieval, specifically for serial collection of cfDNA. EV detection in liquid biopsy is a promising tool for monitoring immunotherapeutic response non-invasively and providing richer information on the parenchymal TME without biopsy across solid tumors (277). In GBM, communication between tumor cells via EV are thought to contribute to the GBM heterogeneity (278). EV biomarkers therefore enable non-invasive monitoring of adaptive responses to GBM immunotherapy while capturing the underlying intratumoral heterogeneity (42). Similarly, quantification and phenotypic analysis of CTC in liquid biopsies is a novel and promising method of characterizing GBM therapeutic response through CSF collection (279, 280). Exploratory research has shown CTC quantification as a plausible prognostic biomarker in GBM through CTC isolation with spiral microfluidics from patient blood samples (281). At present, no studies have established the clinical validity or utility of CTC quantification and analysis for predicting immunotherapy response in GBM. Nonetheless, CTCs represent an attractive noninvasive biomarker platform, as they enable single-cell–level characterization of tumor biology (279).

Moreover, progress in spatial multiomics demonstrates capabilities to identify immunogenic biomarkers despite intratumoral heterogeneity that further elucidate biomarker signatures that are cell-specific and relevant to the TIME cellular landscape (282, 283). Spatial multiomics provides observations of cell-to-cell interactions without losing insights into the tumor architecture and how malignant cells interact in their immediate microenvironment (284, 285). In doing so, spatial multiomics approaches in GBM overcome previous barriers from single-cell RNA sequencing (70). Leveraging spatial multiomics for biomarker discovery is currently in the early stages. A first-of-its-kind study utilized deep learning models to correlate spatial cellular architecture and prognosis by deconvoluting tissue samples into transcriptional subtypes or aggressiveness scores based on gene expression (282). The application of spatial multiomics for predicting biomarker responsiveness remains premature for clinical trial implementation; however, this approach directly addresses the central challenge of intratumoral heterogeneity in GBM.

These innovative techniques facilitate precision medicine in GBM therapy that leverage discovered biomarkers for improved patient selection in clinical trials, especially when organized through AI and machine learning algorithms. AI-integrated clinical trials designs have been explored in the literature for screening and triaging participants (286), prognostication (287), and decision support (288) but are currently inadequately reported or protected from bias (286, 287). Like any other emerging disruption to GBM SOC (289, 290), the integration of AI into clinical trials for GBM immunotherapy requires considerable consensus and regulation with advancing precision oncology in GBM requiring greater standardization and interoperability across computation platforms (291).

## Conclusion

Despite efforts to combat inherent immunotherapy resistance in GBM, current therapeutic lines have not been efficacious in Phase III clinical trials. Still, a poorly identified, small subset of patients with long-term responsiveness to immunotherapy has been observed. Across treatment modalities, biomarkers ranging from peripheral lymphocyte populations to radiogenomic signatures have been proposed; however, biomarkers remain inconsistently validated across cohorts with no standard recommendations for biomarker discovery or implementation in clinical trials. Accordingly, future progress in GBM immunotherapy biomarker development will require several key priorities: (1) international society consensus on biomarker discovery pipelines from exploratory analyzes to investigation in clinical trials, (2) standardization of trial design processes for biomarker identification including assay platforms, endpoints, and statistical analysis, (3) further development of serial collection of liquid biopsies in clinical trials, and (4) prospective validation of multimodal biomarker signatures. While computational approaches may support integration of these data, their role should remain complementary to biologically grounded and clinically validated biomarker strategies. Ultimately, meaningful progress in GBM immunotherapy will depend on the standardization of biomarker discovery pipelines and the coordinated efforts of multidisciplinary teams to develop and validate clinically actionable biomarkers.

## Supplementary material

Supplementary Table 1 Full ClinicalTrials.gov extraction of glioblastoma immunotherapy trials and biomarker characteristics.
