# Supplementary material for: Biomarkers for predicting immunotherapy response and resistance in glioblastoma
Source: Front Immunol. 2026 May 5;17:1823338. doi: 10.3389/fimmu.2026.1823338 (PMC13183666; doi:10.3389/fimmu.2026.1823338)
Supplement: Supplementary Table 1 — Full ClinicalTrials.gov extraction of glioblastoma immunotherapy trials and biomarker characteristics. [file Supplementaryfile1.docx]

Supplementary Table S1. Full ClinicalTrials.gov Extraction of Glioblastoma Immunotherapy Trials and Biomarker Characteristics

| NCT Number | Study Title | Biomarkers Studied | Study Status | Brief Summary | Conditions | Phases | Enrollment | Intervention | Samples Studied | Cell-based Biomarkers | Radiographic Biomarkers | Cytokine Biomarkers | Protein/Molecular Biomarkers | Genetic Biomarkers | Other Biomarkers |
| --- | --- | --- | --- | --- | --- | --- | --- | --- | --- | --- | --- | --- | --- | --- | --- |
| NCT02208362 | Genetically Modified T-cells in Treating Patients With Recurrent or Refractory Malignant Glioma | T-cell levels and phenotype and cytokine levels in tumor cyst fluid, peripheral blood, and cerebrospinal fluid. Tumor levels of IL13Rα2 antigen expression levels at resection and autopsy. | Active but not recruiting | This phase I trial studies the side effects and best dose of genetically modified T-cell immunotherapy in treating patients with malignant glioma that has come back (recurrent) or has not responded to therapy (refractory). A T cell is a type of immune cell that can recognize and kill abnormal cells in the body. T cells are taken from the patient's blood and a modified gene is placed into them in the laboratory and this may help them recognize and kill glioma cells. Genetically modified T-cells may also help the body build an immune response against the tumor cells. | Recurrent Glioblastoma\|Recurrent Malignant Glioma\|Recurrent WHO Grade II Glioma\|Recurrent WHO Grade III Glioma\|Refractory Glioblastoma\|Refractory Malignant Glioma\|Refractory WHO Grade II Glioma\|Refractory WHO Grade III Glioma | PHASE1 | 65 | Adoptive Cell Immunotherapy | Tumor Cyst Fluid, Peripheral Blood, Cerebrospinal fluid, Histology | Yes | None | None | Yes | None | None |
| NCT02455557 | SurVaxM Vaccine Therapy and Temozolomide in Treating Patients With Newly Diagnosed Glioblastoma | Immune Responses to SurVaxM (not described). | Active but not recruiting | This phase II trial studies the side effects and how well vaccine therapy works when given together with temozolomide in treating patients with newly diagnosed glioblastoma. Vaccines made from the survivin peptide or antigen may help the body build an effective immune response to kill tumor cells that express survivin. Drugs used in chemotherapy, such as temozolomide, work in different ways to stop the growth of tumor cells, either by killing the cells, by stopping them from dividing, or by stopping them from spreading. It is not yet known whether temozolomide is more effective with or without vaccine therapy in treating glioblastoma. | Glioblastoma\|Gliosarcoma | PHASE2 | 66 | Vaccine Immunotherapy | *Unknown* | *Unknown* | *Unknown* | *Unknown* | *Unknown* | *Unknown* | *Unknown* |
| NCT02649582 | Adjuvant Dendritic Cell-immunotherapy Plus Temozolomide in Glioblastoma Patients | Cell subset distribution and activation status and antigen-specific immunity in blood | Active but not recruiting | In this phase I/II trial, the primary objective is to determine overall and progression-free survival of patients with newly diagnosed glioblastoma when autologous Wilms' tumor 1 (WT1) messenger (m)RNA-loaded dendritic cell (DC) vaccination is added to adjuvant temozolomide maintenance treatment following (sub)total resection and temozolomide-based chemoradiation. | Glioblastoma Multiforme of Brain | PHASE1\|PHASE2 | 20 | Vaccine Immunotherapy | Peripheral Blood | Yes | None | Yes | Yes | None | None |
| NCT03152318 | A Study of the Treatment of Recurrent Malignant Glioma With rQNestin34.5v.2 | MRI alterations of permeability in injected sites using standard perfusion sequences and MRI alterations of cerebral blood volume in injected sites using standard sequences. | Active recruitment | This research study is evaluating an investigational drug, an oncolytic virus called rQNestin34.5v.2. This research study is a Phase I clinical trial, which tests the safety of an investigational drug and also tries to define the appropriate dose of the investigational drug as a possible treatment for this diagnosis of recurrent or progressive brain tumor. | Malignant Glioma of Brain\|Astrocytoma\|Malignant Astrocytoma\|Oligodendroglioma\|Anaplastic Oligodendroglioma of Brain (Diagnosis)\|Mixed Oligo-Astrocytoma\|Ependymoma\|Ganglioglioma\|Pylocytic/Pylomyxoid Astrocytoma\|Brain Tumor\|Glioma\|Brain Cancer\|Glioblastoma\|Glioblastoma Multiforme | PHASE1 | 62 | Oncolytic Virotherapy | None | None | Yes | None | None | None | None |
| NCT03170141 | Immunogene-modified T (IgT) Cells Against Glioblastoma Multiforme | Production of specific immune check point antibodies in peripheral blood will be measured by ELISA. | Invitation only | This study aims to treat patients who have been diagnosed with brain cancer glioblastoma multiforme (GBM) including diffuse intrinsic pontine glioma (DIPG) and diffuse midline glioma (DMG). The treatment combines two different approaches to fight cancer: immune modulators and antigen-specific T cells. Immune checkpoint antibodies have been tested on various tumors with good outcomes. GBM is known to express increased levels of certain antigens that can be targeted by T cells including chimeric antigen receptor-modified T (CAR-T) cells and tumor antigen specific cytotoxic lymphocytes (CTLs). In this study, the gene-modified T cells specific for GBM antigens will be combined with immune modulatory gene-modified dendritic cells (DCs) as individualized treatment regimens to treat patients. | Glioblastoma Multiforme of Brain\|Brain Cancer | PHASE1 | 30 | Adoptive Cell Immunotherapy | Peripheral Blood | None | None | None | Yes | None | None |
| NCT03382977 | Study to Evaluate Safety, Tolerability, and Optimal Dose of Candidate GBM Vaccine VBI-1901 in Recurrent GBM Subjects | Assessment of IgG antibody to HCMV gB antigen by ELISA and assessment of IFN-γ and IL-5 positive peripheral blood mononuclear cells by ELISPOT. | Active recruitment | The purpose of this study is to assess the safety and tolerability of VBI-1901 in subjects with recurrent malignant gliomas (glioblastoma, or GBM). | Glioblastoma Multiforme | PHASE1\|PHASE2 | 98 | Vaccine Immunotherapy | Peripheral Blood | Yes | None | Yes | Yes | None | None |
| NCT03389230 | Memory-Enriched T Cells in Treating Patients With Recurrent or Refractory Grade III-IV Glioma | Tumor cyst fluid, peripheral blood, and cerebrospinal fluid cytokine levels and HER2 antigen expression levels in tumor tissue. | Active but not recruiting | This phase I trial studies the side effects and best dose of memory-enriched T cells in treating patients with grade II-IV glioma that has come back (recurrent) or does not respond to treatment (refractory). Memory enriched T cells such as HER2(EQ)BBζ/CD19t+ T cells may enter and express its genes in immune cells. Immune cells can be engineered to kill glioma cells in the laboratory by inserting a piece of deoxyribonucleic acid (DNA) into the immune cells that allows them to recognize glioma cells. A vector called lentivirus is used to carry the piece of DNA into the immune cell. It is not known whether these immune cells will kill glioma tumor cells when given to patients. | Glioblastoma\|Malignant Glioma\|Recurrent Glioma\|Refractory Glioma\|WHO Grade III Glioma | PHASE1 | 29 | Adoptive Cell Immunotherapy | Tumor Cyst Fluid, Peripheral Blood, Cerebrospinal fluid, Histology | None | None | Yes | Yes | None | None |
| NCT03491683 | INO-5401 and INO-9012 Delivered by Electroporation (EP) in Combination With Cemiplimab (REGN2810) in Newly-Diagnosed Glioblastoma (GBM) | Change from baseline in Interferon-gamma Secreting T Lymphocytes in PBMCs, change from baseline in T-Cell Phenotypes in PBMCs, change from baseline in T Cell Receptor subtypes in PBMCs, and change from baseline in Antigen-Specific Humoral Response. | Active but not recruiting | Phase 1/2 trial to evaluate safety, immunogenicity and preliminary efficacy of INO-5401 and INO-9012 in combination with cemiplimab (REGN2810), with radiation and chemotherapy, in subjects with newly-diagnosed glioblastoma (GBM). | Glioblastoma | PHASE1\|PHASE2 | 52 | Adoptive Cell Immunotherapy | Peripheral Blood | Yes | None | None | Yes | None | None |
| NCT03548571 | Dendritic Cell Immunotherapy Against Cancer Stem Cells in Glioblastoma Patients Receiving Standard Therapy | Immunological response measured by delayed type hypersensitivity reaction in skin and lymphocyte clonal analysis. | Active recruitment | Open, randomized study of a trivalent dendritic cell therapy compared to standard therapy in primary treated patients with IDH wild-type, MGMT-promotor methylated glioblastoma. The IMP is dendritic cells transfected with mRNA of survivin, hTERT og autologous tumor stem cells derived from tumorspheres. | Glioblastoma | PHASE2\|PHASE3 | 60 | Vaccine Immunotherapy | Tumor | Yes | None | None | None | None | Yes |
| NCT03657576 | Trial of C134 in Patients With Recurrent GBM | White blood cell subset analysis by FACS and intracellular lymphocyte interferon levels will be assessed by FACS analysis ng/mL. | Active but not recruiting | The purpose of this project is to obtain safety information in small groups of individuals, scheduled to receive escalating doses of C134, a cancer killing virus (HSV-1) that has been genetically engineered to safely replicate and kill glioma tumor cells. Safety will be assessed at each dose level before proceeding to the next dose level. A special statistical technique called the Continual Reassessment Method (CRM) will be used to determine when higher doses of virus can be administered. Other objectives of the study include characterization of the activity of C134 after inoculation into the tumor and of the local and systemic immune responses to C134. Patients will also be followed with MRI scans for potential clinical response to C134. The clinical strategy takes advantage of the virus' ability to infect and kill tumor cells while making new virus within the tumors cells; a critical enhancement of this effect is accomplished by the induction of an anti-tumor immune response; both effects are produced by the IRS-1 gene that was placed into the virus by genetic engineering. An additional important component of the research are systematic assessments of the quality of life on treated patients. | Glioblastoma Multiforme of Brain\|Anaplastic Astrocytoma of Brain\|Gliosarcoma of Brain | PHASE1 | 19 | Oncolytic Virotherapy | Peripheral Blood | Yes | None | Yes | None | None | None |
| NCT03688178 | DC Migration Study to Evaluate TReg Depletion In GBM Patients With and Without Varlilumab | Median percent change between baseline, assessed on day 14, and nadir levels of Treg before the time that the second cycle of adjuvant TMZ would be administered and median Chemokine (C-C motif) ligand 3 (CCL3) Levels in Serum at 24, 48, and 72 hours after Pre-conditioning. | Active but not recruiting | Patients with newly diagnosed glioblastoma will be consented following tumor resection then undergo leukapheresis for harvest of peripheral blood leukocytes for generation of dendritic cells. Subjects will then receive standard of care (planned 6 weeks) radiation therapy (RT) and concurrent temozolomide (TMZ) at a standard targeted dose of 75 mg/m2/day.  The study cycle of TMZ comprises a targeted dose of 150-200mg/m2/day for 5 days every 4 (+2) weeks for up to 12 cycles (patients with unmethylated MGMT gene promoter will receive only cycle 1). All patients will receive up to a total of 10 DC vaccines called pp65 CMV dendritic cells (DC). Dendritic Cell (DC) vaccines #1-3 will be given every two weeks, thus delaying the initiation of TMZ cycle 2 for patients receiving TMZ. All remaining TMZ/vaccine cycles will be 4 (+2) weeks in length.  After the first 3 DC vaccines given during Cycle 1 of TMZ, the remaining DC vaccine injections are given on Day 21 (+/- 2 days) of each TMZ cycle. Subjects with unmethylated MGMT will only receive one cycle of adjuvant TMZ; however, their vaccine schedule will follow the same 4 (+ 2) week TMZ cycle schedule.  Following RT, patients will be randomized into 1 of 3 groups. Groups 1 and 2 will be blinded. The groups differ in the type of pre-conditioning received prior to DC vaccine #4; additionally, Group 3 will be receiving infusions of varlilumab 7 days prior to and with vaccine #1 and 7 days prior to vaccine #3+. The pre-conditioning for each group is as follows: Group 1: Unpulsed DC pre-conditioning prior to DC vaccine #4; Group 2: Tetanus-diphtheria (Td) pre-conditioning prior to DC vaccine #4; Group 3: Td pre-conditioning prior to DC vaccine #4 and varlilumab infusion at 7 days prior to each DC vaccine (except DC vaccine #2) with Td pre-conditioning prior to vaccine #4. | Glioblastoma | PHASE2 | 43 | Novel Immunotherapy | Peripheral Blood | Yes | None | Yes | None | None | None |
| NCT04003649 | IL13Ra2-CAR T Cells With or Without Nivolumab and Ipilimumab in Treating Patients With GBM | CAR T and endogenous T cell levels and phenotype detected in and cytokine levels in tumor cyst fluid, peripheral blood, and cerebral spinal fluid. Area under the curve (AUC) for CD3, IFNgamma, and IP-10 levels over time. CAR T and endogenous cells detected in, IL13Ralpha2 antigen expression levels in, and PD-L1 levels on tumor cells in tumor tissue. | Active recruitment | This phase I trial studies the side effects and how well IL13Ralpha2-CAR T cells work when given alone or together with nivolumab and ipilimumab in treating patients with glioblastoma that has come back (recurrent) or does not respond to treatment (refractory). Biological therapies, such as IL13Ralpha2-CAR T cells, use substances made from living organisms that may attack specific glioma cells and stop them from growing or kill them. Immunotherapy with monoclonal antibodies, such as nivolumab and ipilimumab, may help the body's immune system attack the cancer, and may interfere with the ability of tumor cells to grow and spread. It is not yet known whether giving IL13Ralpha2-CAR T cells and nivolumab together may work better in treating patients with glioblastoma. | Recurrent Glioblastoma\|Refractory Glioblastoma | PHASE1 | 60 | CAR T-Cell Immunotherapy | Tumor Cyst Fluid, Peripheral Blood, Cerebrospinal fluid, Histology | Yes | None | Yes | Yes | None | None |
| NCT04015700 | Neoantigen-based Personalized DNA Vaccine in Patients With Newly Diagnosed, Unmethylated Glioblastoma | Immunogenicity measured as T-cell phenotype, myeloid derived suppressor cell frequency by flow cytometry, diversity of clonality from T cell receptor sequencing, and putative antigen specificity from T cell receptor sequencing. | Active but not recruiting | This is a single institution, open-label, single arm, study assessing the safety, feasibility, and immunogenicity of a personalized neoantigen-based vaccine in subjects with newly diagnosed, unmethylated glioblastoma. | Glioblastoma | PHASE1 | 9 | Vaccine Immunotherapy | Tumor | Yes | None | None | Yes | None | None |
| NCT04201873 | Pembrolizumab and a Vaccine (ATL-DC) for the Treatment of Surgically Accessible Recurrent Glioblastoma | Biomarker analysis pre- and post-treatment (unspecified), TIL density and TCR clonality in the tumor quantitatively and in the peripheral blood, gene expression signature and somatic mutations in the tumor and blood measured by RNA Seq and nano string IO360, T cell subset and activation markers within peripheral blood measured by flow cytometry, and TIL quantification including tumor quantification of PD-1, PD-L1, CD3, CD4, CD8, Iba-I, Ki-67 measured by immunohistochemistry of FFPE tissue. | Active but not recruiting | This phase I trial studies the side effects and how well of pembrolizumab and a vaccine therapy (ATL-DC vaccine) work in treating patients with glioblastoma that has come back (recurrent) and can be removed by surgery (surgically accessible). Immunotherapy with monoclonal antibodies, such as pembrolizumab, may help the body's immune system attack the cancer, and may interfere with the ability of tumor cells to grow and spread. Vaccines, such as ATL-DC vaccine, may help the body build an effective immune response to kill tumor cells. Giving pembrolizumab and ATL-DC vaccine may work better in treating patients with glioblastoma compared to ATL-DC alone. | Recurrent Glioblastoma | PHASE1 | 40 | Vaccine Immunotherapy | Tumor, Peripheral Blood, Histology | Yes | None | None | Yes | Yes | None |
| NCT04214392 | Chimeric Antigen Receptor (CAR) T Cells With a Chlorotoxin Tumor-Targeting Domain for the Treatment of MMP2+ Recurrent or Progressive Glioblastoma | T-cell levels and phenotype and cytokine levels in tumor cyst fluid, peripheral blood, and cerebrospinal fluid. Chlorotoxin-targeted antigen expression levels in tumor tissue. | Active but not recruiting | This phase I trial studies the side effects and best dose of chimeric antigen receptor (CAR) T cells with a chlorotoxin tumor-targeting domain in treating patients with MPP2+ glioblastoma that has come back (recurrent) or that is growing, spreading, or getting worse (progressive). Vaccines made from a gene-modified virus may help the body build an effective immune response to kill tumor cells. | Recurrent Glioblastoma\|Recurrent Malignant Glioma\|Recurrent WHO Grade II Glioma\|Recurrent WHO Grade III Glioma | PHASE1 | 19 | CAR T-Cell Immunotherapy | Tumor Cyst Fluid, Peripheral Blood, Cerebrospinal fluid, Histology | Yes | None | None | Yes | None | None |
| NCT04323046 | Immunotherapy Before and After Surgery for Treatment of Recurrent or Progressive High Grade Glioma in Children and Young Adults | Relative changes in interferon gamma associated genetic signature within the tumor microenvironment post administration of neoadjuvant nivolumab, correlation of interferon-gamma-associated genetic signature, cell cycle-related genetic signature and infiltrating T lymphocyte (TIL) density and clonality with clinical responses, TIL density post administration of neoadjuvant nivolumab, association between advanced MRI parameters (ADC on DWI, rCBV on dynamic susceptibility contrast (DSC) perfusion MRI, pre-contrast T1 shortening on T1-weighed images, and/or MTRasym on pH-Weighted Amine CEST-EPI) and tumor and peripheral blood immune responses, relative change in peripheral T-cell response and post administration of neo-adjuvant nivolumab, measurement of PD-1 and PDL-1 expression by immunohistochemistry, and the correlation of tumor mutational load with clinical response. | Active recruitment | This phase I trial studies the side effects of nivolumab before and after surgery in treating children and young adults with high grade glioma that has come back (recurrent) or is increasing in scope or severity (progressive). Immunotherapy with monoclonal antibodies, such as nivolumab, may help the body's immune system attack the cancer, and may interfere with the ability of tumor cells to grow and spread. | Glioblastoma\|Malignant Glioma\|Recurrent Glioblastoma\|Recurrent Malignant Glioma\|Recurrent Grade III Glioma\|Grade III Glioma | PHASE1 | 20 | Immune Checkpoint Blockade | Tumor, Peripheral Blood, Histology | Yes | Yes | None | Yes | Yes | None |
| NCT04485949 | A Phase 2b Clinical Study With a Combination Immunotherapy in Newly Diagnosed Patients With Glioblastoma | Clinically Significant Laboratory Assessment Abnormalities, Clinically Significant Vital Signs Measurements, and Clinically Significant Physical Examination Findings. | Active but not recruiting | The purpose of this study is to assess progression-free survival (PFS) and overall survival (OS) in newly diagnosed Glioblastoma (GBM) participants treated with IGV-001 as compared with placebo. | Glioblastoma | PHASE2 | 93 | Adoptive Cell Immunotherapy | *Unknown* | Yes | None | Yes | None | None | None |
| NCT04523688 | Vaccination With Autologous Dendritic Cells Loaded With Autologous Tumour Homogenate in Glioblastoma | Evaluation of the prognostic role of a positive delayed hypersensitivity skin test after at least four vaccine administrations, HLA class I and II characterization of patients, prognostic and predictive role of tumor antigen expression in tumor tissue and the prognostic and predictive role of immune cells in the peripheral blood and in the tumor microenvironment. | Active recruitment | Single arm, monocentric trial to assess the safety and the progression-free survival related to the combined treatment of dendritic cell vaccine loaded with autologous tumor homogenate and temozolomide in patients operated for glioblastoma and then treated with standard radiochemotherapy (according to Stupp regimen). | Glioblastoma\|Vaccination | PHASE2 | 28 | Vaccine Immunotherapy | Tumor, Peripheral Blood | Yes | None | None | Yes | None | Yes |
| NCT04656535 | AB154 Combined With AB122 for Recurrent Glioblastoma | Single cell RNA sequencing of tumor and blood after exposure to AB154 with and without AB122 and Tregs and CD8 T cells ratio by immunofluorescence. | Active but not recruiting | This is a phase 0/I exploratory study. Patients at first or second recurrence of glioblastoma will be enrolled. The study will be divided into two cohorts: Cohort A (safety cohort) and Cohort B (surgical patient cohort).  Cohort A: Eligible patients will be sequentially enrolled to receive intravenous domvanalimab combined with zimberelimab (N=6). Domvanalimab will be given at a dose of 10 mg/kg and zimberelimab will be given at a dose of 240 mg (flat). The dosing was determined in a separate study in solid tumors; this cohort will confirm the safety of the dosing schedule in patients with brain tumors.  Cohort B: Expansion surgical cohort. The purpose of cohort B is to provide an additional safety evaluation of domvanalimab + zimberelimab as well as tissue and blood for exploratory ancillary studies investigating the effects of domvanalimab + zimberelimab in the tumor and tumor microenvironment. A total of 46 patients will be enrolled in this cohort. | Glioblastoma | EARLY_PHASE1 | 46 | Immune Checkpoint Blockade | Tumor, Peripheral Blood | Yes | None | None | None | Yes | None |
| NCT04661384 | Brain Tumor-Specific Immune Cells (IL13Ralpha2-CAR T Cells) for the Treatment of Leptomeningeal Glioblastoma, Ependymoma, or Medulloblastoma | IL13Ralpha2-CAR T cell persistence in the tumor tissue and the location of the IL13Ralpha2-CAR T cells with respect to the infusion site. IL13Ralpha2 antigen on tumor tissue pre- and post-CAR T cell therapy. Cytokine levels in tumor cyst fluid, peripheral blood, and cerebrospinal fluid. | Active but not recruiting | This phase I trial investigates the side effects of brain tumor-specific immune cells (IL13Ralpha2-CAR T cells) in treating patients with leptomeningeal disease from glioblastoma, ependymoma, or medulloblastoma. Immune cells are part of the immune system and help the body fight infections and other diseases. Immune cells can be engineered to destroy brain tumor cells in the laboratory. IL13Ralpha2-CAR T cells is brain tumor specific and can enter and express its genes in immune cells. Giving IL13Ralpha2-CAR T cells may better recognize and destroy brain tumor cells in patients with leptomeningeal disease from glioblastoma, ependymoma or medulloblastoma. | Ependymoma\|Glioblastoma\|Medulloblastoma\|Recurrent Metastatic Malignant Neoplasm in the Leptomeninges | PHASE1 | 10 | CAR T-Cell Immunotherapy | Tumor Cyst Fluid, Peripheral Blood, Cerebrospinal fluid, Histology | None | None | Yes | Yes | None | None |
| NCT04801147 | Immunotherapy With Autologous Tumor Lysate-Loaded Dendritic Cells In Patients With Newly Diagnosed Glioblastoma Multiforme | Immune response will be monitored throughout study during active treatment at least on vaccination-time points and later an average of 2 months. | Active recruitment | Rationale of the Study: Treatment for GBM currently consists of surgical resection of the tumour mass followed by radio- and chemotherapy ((1)Stupp et al., 2005). Nonetheless overall prognosis still remains bleak, recurrence is universal, and recurrent GBM patients clearly need innovative therapies. Dendritic cells (DC) immunotherapy could represent a well-tolerated, long-term tumour-specific treatment to kill all (residual) tumour cells which infiltrate in the adjacent areas of the brain. Preclinical investigations for the development of therapeutic vaccines against high grade gliomas, based on the use of DC loaded with a mixture of glioma-derived tumor have been carried out in rat as well as in mouse models, showing the capacity to generate a glioma-specific immune response. Mature DC loaded with autologous tumor lysate have been used also for the treatment of patients with recurrent malignant brain tumors; no major adverse events have been registered. Results about the use of immunotherapy for GBM patients are encouraging, but further studies are necessary to find out the most effective and safe combination of immunotherapy with radio- and chemotherapy after exeresis of the tumour mass.  Aim of the study. Primary objective of the study is to evaluate treatment tolerability and to get preliminary information about efficacy. Secondary objective is to evaluate the treatment effect on the immune response. Additional objective is to identify a possible correlation between methylation status of MGMT promoter and tumor response to treatment.  A two-stage Simon design ((2)Simon, 1989) will be considered for the study. Assuming as outcome measure the percentage of PFS12 patients and of clinical interest an increase to 42% (P1) of the historical control rate of 27% (P0) ((1)Stupp et al., 2005), the alternative hypothesis will be rejected at the end of the first stage if the PFS12 rate will be less than 8/24 treated patients (Fisher's exact test). In the second stage patients will be enrolled up to 76 overall. The null hypothesis will be rejected (a=0.05, b=0.2) if at least 27 subjects out of 76 are alive and progression free 12 months after the beginning of the treatment. | Glioblastoma | PHASE1\|PHASE2 | 76 | Vaccine Immunotherapy | Tumor | Yes | None | Yes | None | None | None |
| NCT04808245 | A MultIceNTER Phase I Peptide VaCcine Trial for the Treatment of H3-Mutated Gliomas | Immunogenicity in response to treatment mostly defined as T cell responses are measured on PBMCs using IFN-gamma ELISpot. | Active but not recruiting | The study "A MultIceNTER Phase I Peptide VaCcine Trial to Exploit NeoePitope-Specific T Cells for the Treatment of H3K27M-Mutated Gliomas - (INTERCEPT H3)" is a non-controlled, open-label, single arm, multicenter phase I trial involving patients with gliomas carrying an H3.1K27M or H3.3K27M mutation. | Newly Diagnosed H3-mutated Glioma | PHASE1 | 15 | Vaccine Immunotherapy | Peripheral Blood | Yes | None | Yes | None | None | None |
| NCT04968366 | Safety & Efficacy of DC Vaccine and TMZ for the Treatment of Newly-diagnosed Glioblastoma After Surgery | Peripheral tumor specific immune response measured by FN-γ release ELISPOT assay, and the peripheral blood mononuclear cells (PBMCs) that are collected before the vaccine injection (baseline), one week after and at the 3r, 5th and the last injection. | Active but not recruiting | This is a single-center, single-arm phase I study to determine the safety and preliminary efficacy of autologous dendritic cells (DCs) loaded with multiple tumor neoantigen peptides administered as a cancer-treatment vaccine to treat adult postoperative patients with newly-diagnosed glioblastoma, in combination with the standard-of-care Temozolomide (TMZ) chemotherapy. | Glioblastoma Multiforme of Brain | PHASE1 | 11 | Vaccine Immunotherapy | Peripheral Blood | Yes | None | Yes | None | None | None |
| NCT04977375 | Trial of Anti-PD-1 Immunotherapy and Stereotactic Radiation in Patients With Recurrent Glioblastoma | Assess the T cell clonality, CD8 T cell activation and Tumor Infiltrating Lymphocyte (TIL) score after treatment. Blood draws are prior to neoadjuvant pembrolizumab administration and at the time of initiation of SRT and at the time of surgery | Active recruitment | The purpose of this study is to assess the safety/tolerability/feasibility of pembrolizumab and radiation therapy before surgical resection in patients with recurrent glioblastoma as defined by treatment-related AEs and the number of patients who do not necessitate a delay in surgical resection, and to assess overall survival. The secondary objectives are to assess progression free survival, and to assess the T cell clonality, CD8 T cell activation and Tumor Infiltrating Lymphocyte (TIL) score after treatment | Glioblastoma Multiforme | PHASE1\|PHASE2 | 10 | Immune Checkpoint Blockade | Tumor, Peripheral Blood | Yes | None | None | None | None | None |
| NCT05139056 | Multiple Intracerebral Doses of Neural Stem Cell-Based Virotherapy (NSC-CRAd-S-pk7) for the Treatment of Recurrent High-Grade Gliomas | Changes in survivin expression by immunohistochemistry IHC in pre- and post-treatment tissue, changes in immune cell populations in the tumor microenvironment in pre- and post-treatment tumor tissue samples assessed by Vectra Spectral Imaging, and multiplex immunoassays in cerebrospinal fluid (CSF) samples and RNA-sequencing in CSF, peripheral blood, and tumor tissue samples. | Active recruitment | This phase I trial studies the safety of giving multiple intracerebral doses of NSC-CRAd-S-pk7 to treat patients with glioblastoma at first recurrence. NSC-CRAd-S-pk7 consists of neural stem cells that can target glioblastoma cells and carry a virus, which can kill cancer cells. Giving multiple doses of NSC-CRAd-S-pk7 may kill more tumor cells. | Currently Only Enrolling Glioblastoma Patients at First Recurrence | PHASE1 | 36 | Oncolytic Virotherapy | Tumor, Peripheral Blood, Cerebrospinal fluid, Histology | Yes | Yes | Yes | Yes | Yes | None |
| NCT05163080 | SurVaxM Plus Adjuvant Temozolomide for Newly Diagnosed Glioblastoma (SURVIVE) | Predictive value of perfusion-weighted imaging, objective image based tumor response rate, and molecular predictors of response to SurVaxM including MGMT methylation status, anti-surviving immunoglobin titers, surviving-specific CD8+ responses, tumor survivin expression levels and other molecular tumor tissue markers. | Active but not recruiting | The main purpose of this study is to determine whether adding SurVaxM to standard-of-care temozolomide chemotherapy is better than temozolomide treatment alone for patients with newly diagnosed glioblastoma. This study is designed to compare the length of survival in patients with newly diagnosed glioblastoma who receive temozolomide plus SurVaxM to that of patients treated with standard-of-care temozolomide plus placebo. This study aims to discover what effects, both good and bad, this combination of drugs may have on you and to see if the study drug (SurVaxM) can create an immune response in your blood that is directed against your cancer cells. This study also aims to determine whether treatment with SurVaxM plus temozolomide improves the survival of glioblastoma patients like yourself compared to treatment with temozolomide alone. | Newly Diagnosed Glioblastoma | PHASE2 | 247 | Vaccine Immunotherapy | Tumor | Yes | Yes | Yes | Yes | None | None |
| NCT05235737 | The Assessment of Immune Response in Newly Diagnosed Glioblastoma Patients Treated With Pembrolizumab | Usability assessment of immuno-PET imaging with 89Zr-DFO-Atezolizumab for quantitative analysis of early changes in PD-L1 expression and T-cell levels. | Active recruitment | To evaluate the short-term and longer-term safety, tolerability, and effectiveness of neoadjuvant and adjuvant Pembrolizumab on top of standard therapy (Stupp protocol) in patients with Glioblastoma Multiforme (GBM).  Randomized comparison of safety, tolerability, and clinical efficacy of (1) neoadjuvant and adjuvant Pembrolizumab (on top of Stupp protocol, n=12 patients), (2) neoadjuvant Pembrolizumab (on top of Stupp protocol, n=12 patients), and (3) standard of care (Stupp protocol only, n=12 patients). Immuno-PET examination will be performed before and after surgery in all patients. | Newly Diagnosed Glioblastoma | PHASE4 | 36 | Immune Checkpoint Blockade | Tumor | Yes | Yes | None | Yes | None | None |
| NCT05283109 | ETAPA I: Peptide-based Tumor Associated Antigen Vaccine in GBM | Change in mean fold increase in pp56-specific T cells stratified between CMV seropositive and seronegative patients, change in mean fold increase in EphA2- or survivin- specific T cells, and change in mean fold increase in pp56-specific T cells. | Active but not recruiting | This is a phase 1b study of P30-linked EphA2, CMV pp65, and survivin vaccination (collectively called the P30-EPS vaccine) in HLA-A\*0201 positive patients with a newly diagnosed, unmethylated, and untreated World Health Organization (WHO) grade IV malignant glioma. | Glioma, Malignant | PHASE1 | 24 | Vaccine Immunotherapy | Tumor | Yes | None | None | Yes | None | None |
| NCT05685004 | Study of Neoantigen-specific Adoptive T Cell Therapy for Newly Diagnosed MGMT Negative Glioblastoma Multiforme (GBM) | Immunogenicity measured as delayed-type hypersensitivity skin test 24 hours after each administration and other genetic and immunologic parameters 24 hours after vaccine administration. | Active but not recruiting | This randomized study is designed to compare the combination of TVI-Brain-1 immunotherapy and standard therapy compared to standard therapy alone as a treatment for newly diagnosed MGMT unmethylated glioblastoma patients. The patients' own cancer cells collected after surgery are combined into a vaccine to produce an immune response that significantly increases the number of cancer neoantigen-specific effector T cell precursors in the patient's body. These cancer neoantigen-specific T cells are harvested from the blood, subsequently stimulated and expanded, and infused back into the patient. | Glioblastoma Multiforme of Brain | PHASE2\|PHASE3 | 120 | Adoptive Cell Immunotherapy |  | None | None | None | None | Yes | Yes |
| NCT05698199 | Study to Evaluate the Safety, Tolerability, Immunogenicity and Preliminary Efficacy of ITI-1001 In Patients With Newly Diagnosed Glioblastoma (GBM) | Changes in peripheral blood assessment of T cell activation through ELISpot, flow cytometry, CMV serology and anti-LAMP1 antibody for evaluation of immune response from the baseline, changes in immune response through nalytical evaluation of MRI data for possible tumor relapse or activated immune cells, and changes in immune response through Immunohistochemistry (IHC) evaluation from original biopsy/surgical sample and a second biopsy/surgical procedure if recurrence is suspected, specifically IHC detects cytomegalovirus (CMV) proteins pp65, IE1, and Glycoprotein B (gB) in human glioblastoma multiforme tissues as well as the CMV markers (CD8, CD163, and FOXP3), and changes in Neurological Assessment in Neuro-oncology (NANO) scale from baseline. | Active but not recruiting | This Phase I clinical trial will evaluate the safety, tolerability, immunogenicity, and preliminary efficacy of 8 mg ITI-1001 in participants with newly diagnosed glioblastoma (GBM). | Glioblastoma | PHASE1 | 10 | Vaccine Immunotherapy | Tumor, Peripheral Blood, Histology | Yes | Yes | Yes | Yes | None | None |
| NCT05743595 | Neoantigen-based Personalized DNA Vaccine With Retifanlimab PD-1 Blockade Therapy in Patients With Newly Diagnosed, Unmethylated Glioblastoma | Immunogenicity as measured by the number of subjects in a cohort who develop at least one demonstrable neoantigen CD8 T cell response by day 71 after administration of the first dose of vaccine, as measured by the percentage of neoantigens that elicit a neoantigen-specific CD8 T cell response out of the total number of neoantigens vaccinated against within a cohort, s measured by T-cell phenotype, myeloid derived suppressor cell frequency assessed by flow cytometry, as measured by T cell receptor sequencing to assess diversity of clonality and putative antigen specificity, and as measured by pro- and anti-inflammatory chemokine analysis and cytokine analysis in plasma as assessed by multiplex ELISA. | Active recruitment | This is a single institution, open-label, multi-arm, phase I study assessing the safety and immunogenicity of a personalized neoantigen-based personalized DNA vaccine combined with PD-1 blockade therapy in subjects with newly diagnosed, MGMT promoter unmethylated glioblastoma (GBM).  Immune checkpoint blockade, specifically those targeting the PD-1/PD-L1 pathways, has shown efficacy in multiple solid and hematologic malignancies. Furthermore, as has been demonstrated in metastatic melanoma, combining PD-1/PD-L1 blockade with other immune checkpoint inhibitors has shown improved objective response rates, though there is a significant increase in serious immune-related adverse events. As such, current trials are exploring different doses, administration schedules, and immune checkpoint agents. One alternative approach, however, is to introduce a tumor-directed therapy such as a personalized neoantigen vaccine combined with these immune modulating agents (i.e. immune checkpoint blocking antibodies) to maximize the tumor-specific response but minimize the toxicity associated with increasing non-specific systemic immune activation by generating a potent and focused neoantigen specific immune response.  This study will test the hypothesis that a personalized neoantigen DNA vaccine in combination with concurrent administration of immune checkpoint blockade therapy will enhance the magnitude and breadth of neoantigen-specific T cell responses while maintaining an acceptable safety profile. The overall goal of this study is to identify the optimal vaccine plus adjuvant platform that can be tested in a subsequent phase II study to determine the efficacy of a personalized neoantigen vaccine approach in patients with GBM. | Unmethylated Glioblastoma | PHASE1 | 27 | Vaccine Immunotherapy | Tumor | Yes | None | Yes | Yes | None | None |
| NCT05864534 | Phase 2a Immune Modulation With Ultrasound for Newly Diagnosed Glioblastoma | Phospho-extracellular signal-related kinase (p-ERK) expression, Blood circulating tumor DNA | Active recruitment | Brain tumor treatment is hampered by the blood-brain barrier (BBB). This barrier prevents drugs carried in the bloodstream from getting into the brain. If the BBB can be opened, making it temporarily more permeable, drugs may able to better reach the brain tumor. In this trial we will implant a novel device with 9 ultrasound emitters, allowing temporary and reversible opening of the BBB to maximize brain penetration of drugs that modulate the immune system. The device will be implanted after radiation is completed. Immune modulating drugs will be given every 3 weeks in conjunction with activation of the device to open the BBB.  The objectives of this trial are to establish whether it is safe and feasible to administer immune modulating drugs in this manner, and identify whether the treatment is effective in treating glioblastoma. | Newly Diagnosed Glioblastoma\|Glioblastoma, Isocitric Dehydrogenase (IDH)-Wildtype\|Gliosarcoma\|Glioblastoma Multiforme | PHASE2 | 25 | Novel Immunotherapy | Tumor, Peripheral Blood | None | None | None | Yes | Yes | None |
| NCT06043232 | MMR/MSI Phenotypes in Prediction of Tumor Vaccine Benefit for Gliomas | Transcriptome sequencing to measure gene expression level, TCR/BCR sequencing to measure clonality of lymphocytes, TCR/BCR sequencing to measure gene expression level in protein, features from images, whole genome sequencing or whole exome sequencing to measure gene mutations, and IHC analysis. | Active recruitment | Glioblastoma (GBM) is the most malignant primary intracranial tumor with a median survival of about 18 months, and new therapies are urgently needed. Tumor vaccines has been shown to improve survival of GBM, but not all patients can benefit from vaccine treatment and biomarkers are urgently needed. Deletion of mismatch repair (MMR) protein and microsatellite instability (MSI) state are important features in the biological evolution of GBM, and may be used as markers for tumor vaccine. Therefore, this project will collect samples from GBM patients before and after vaccine treatment respectively, and evaluate the role of MMR/MSI gene phenotype in predicting vaccine efficacy and the potential molecular mechanism. Moreover, MMR/MSI phenotypes will be assessed by deep-learning and radiomics using images to establish noninvasive markers for vaccine. | Glioma |  | 360 | Vaccine Immunotherapy | Tumor | Yes | Yes | Yes | Yes | Yes | None |
| NCT06132438 | Immunotherapy Targeting of Cytomegalovirus Antigens in Glioblastoma | Immunologic response as measured by peak number of T cells that secrete IFNy by ELISPOT in response to component A of PEP-CMV | Not yet recruiting | In Australia, glioblastoma (GBM) has a higher annual fatality rate than a variety of other cancers, such as melanoma, bladder, and kidney tumors. While the 5-year survival rate for other cancers, such as breast and prostate cancer, has increased, there have been no notable advancements in GBM during the past ten years, and the incidence and mortality patterns have barely changed between 1982 and 2011. In particular, GBM poses a challenging therapeutic dilemma for patients and physicians due to its aggressive biology and resistance to available treatments. Recent studies showed that cytomegalovirus (CMV) is expressed in GBM tumors, making it a good target for immunotherapy trials. This phase I trial aims to determine the safety and tolerability of the PEP-CMV vaccine in patients with newly diagnosed MGMT-unmethylated GBM in combination with one cycle of adjuvant temozolomide. | Glioblastoma | PHASE1 | 26 | Vaccine Immunotherapy | Tumor | Yes | None | Yes | None | None | None |
| NCT06749925 | Clinical Trial Assessing the Efficacy and Safety of Dendritic Cell-Based Immunotherapy for Glioblastoma | Immunological response measured as levels of Th1-pattern tumor-reactive T lymphocytes in peripheral blood. | Not yet recruiting | This Phase III, multicenter, placebo-controlled clinical trial with sequential randomization is designed to evaluate the efficacy and safety of an experimental vaccine composed of hybrid dendritic cells (DCs) for the treatment of glioblastoma. Conducted at the Hospital das Clínicas of the University of São Paulo Medical School (HCFMUSP) and the Institute of Biomedical Sciences of the University of São Paulo (ICB/USP), the study is led by Professor José Alexandre Marzagão Barbuto. A multidisciplinary team of researchers specializing in neurosurgery, pathology, hematology, and other fields will contribute to a comprehensive approach.  The trial aims to determine whether the hybrid DC vaccine can increase overall survival in adult patients with glioblastoma who have completed standard treatment, including surgery, chemotherapy, and radiotherapy. Secondary objectives include evaluating progression-free survival, quality of life, immune response, and the safety of the intervention. The study will enroll 186 patients, who will be randomized into three groups: (1) a control group receiving placebo, (2) a group receiving the DC vaccine, and (3) a group receiving the DC vaccine combined with pembrolizumab. | Glioblastoma | PHASE3 | 186 | Vaccine Immunotherapy | Peripheral Blood | Yes | None | None | None | None | None |
| NCT06816927 | Trial of Glioblastoma Immunotherapy Advancement With Nivolumab and Relatlimab | Changes in TILs in formalin-fixed paraffin embedded (FFPE) tumor sections from biopsy to resection during concomitant nivolumab, relatlimab, RT and TMZ. | Not yet recruiting | GIANT is an open-label, multi-center, randomized, perioperative (neoadjuvant followed by adjuvant), phase 2 trial with a safety lead-in phase to investigate the feasibility, safety and tolerability, and establish the biological activity of nivolumab with or without relatlimab in patients with isocitrate dehydrogenase (IDH) wildtype newly diagnosed glioblastoma (ndGBM). | Newly Diagnosed Glioblastoma | PHASE2 | 92 | Immune Checkpoint Blockade | Histology | Yes | None | None | None | None | None |

**Supplementary Table S1. Full ClinicalTrials.gov Extraction of Glioblastoma Immunotherapy Trials and Biomarker Characteristics.** Table contains original extraction from [Clinicaltrials.gov](http://clinicaltrials.gov) on January 04, 2026 utilizing the terms “Glioblastoma” and “immunotherapy OR Vaccine Therapy OR CAR T-cells OR oncolytic virus.” All secondary data collection was interpreted from study descriptions verbatim.
